# Genome-wide expression and network analyses of mutants in key brassinosteroid signaling genes

**DOI:** 10.1186/s12864-021-07778-w

**Published:** 2021-06-22

**Authors:** Razgar Seyed Rahmani, Tao Shi, Dongzhi Zhang, Xiaoping Gou, Jing Yi, Giles Miclotte, Kathleen Marchal, Jia Li

**Affiliations:** 1 Department of Plant Biotechnology and Bioinformatics, Ghent University, Ghent, Belgium; 2 Department of Information Technology, IDLab, imec, Ghent University, Ghent, Belgium; 3 Key Laboratory of Aquatic Botany and Watershed Ecology, Wuhan Botanical Garden, Chinese Academy of Sciences, Wuhan, 430074 China; 4 Ministry of Education Key Laboratory of Cell Activities and Stress Adaptations, School of Life Sciences, Lanzhou University, Lanzhou, 730000 China; 5 Department of Biochemistry, Genetics and Microbiology, University of Pretoria, Pretoria, South Africa

**Keywords:** Brassinosteroid signaling, Expression analysis, Systems biology, Network analysis, Arabidopsis

## Abstract

**Background:**

Brassinosteroid (BR) signaling regulates plant growth and development in concert with other signaling pathways. Although many genes have been identified that play a role in BR signaling, the biological and functional consequences of disrupting those key BR genes still require detailed investigation.

**Results:**

Here we performed phenotypic and transcriptomic comparisons of *A. thaliana* lines carrying a loss-of-function mutation in *BRI1* gene, *bri1–*5, that exhibits a dwarf phenotype and its three activation-tag suppressor lines that were able to partially revert the *bri1–5* mutant phenotype to a WS2 phenotype, namely *bri1–5/bri1–1D*, *bri1–5/brs1–1D,* and *bri1–5/bak1–1D.* From the three investigated *bri1–5* suppressors, *bri1–5/bak1–1D* was the most effective suppressor at the transcriptional level. All three *bri1–5* suppressors showed altered expression of the genes in the abscisic acid (ABA signaling) pathway, indicating that ABA likely contributes to the partial recovery of the wild-type phenotype in these *bri1–5* suppressors. Network analysis revealed crosstalk between BR and other phytohormone signaling pathways, suggesting that interference with one hormone signaling pathway affects other hormone signaling pathways. In addition, differential expression analysis suggested the existence of a strong negative feedback from BR signaling on BR biosynthesis and also predicted that *BRS1*, rather than being directly involved in signaling, might be responsible for providing an optimal environment for the interaction between BRI1 and its ligand.

**Conclusions:**

Our study provides insights into the molecular mechanisms and functions of key brassinosteroid (BR) signaling genes, especially *BRS1*.

**Supplementary Information:**

The online version contains supplementary material available at 10.1186/s12864-021-07778-w.

## Background

**Fig. 1:**
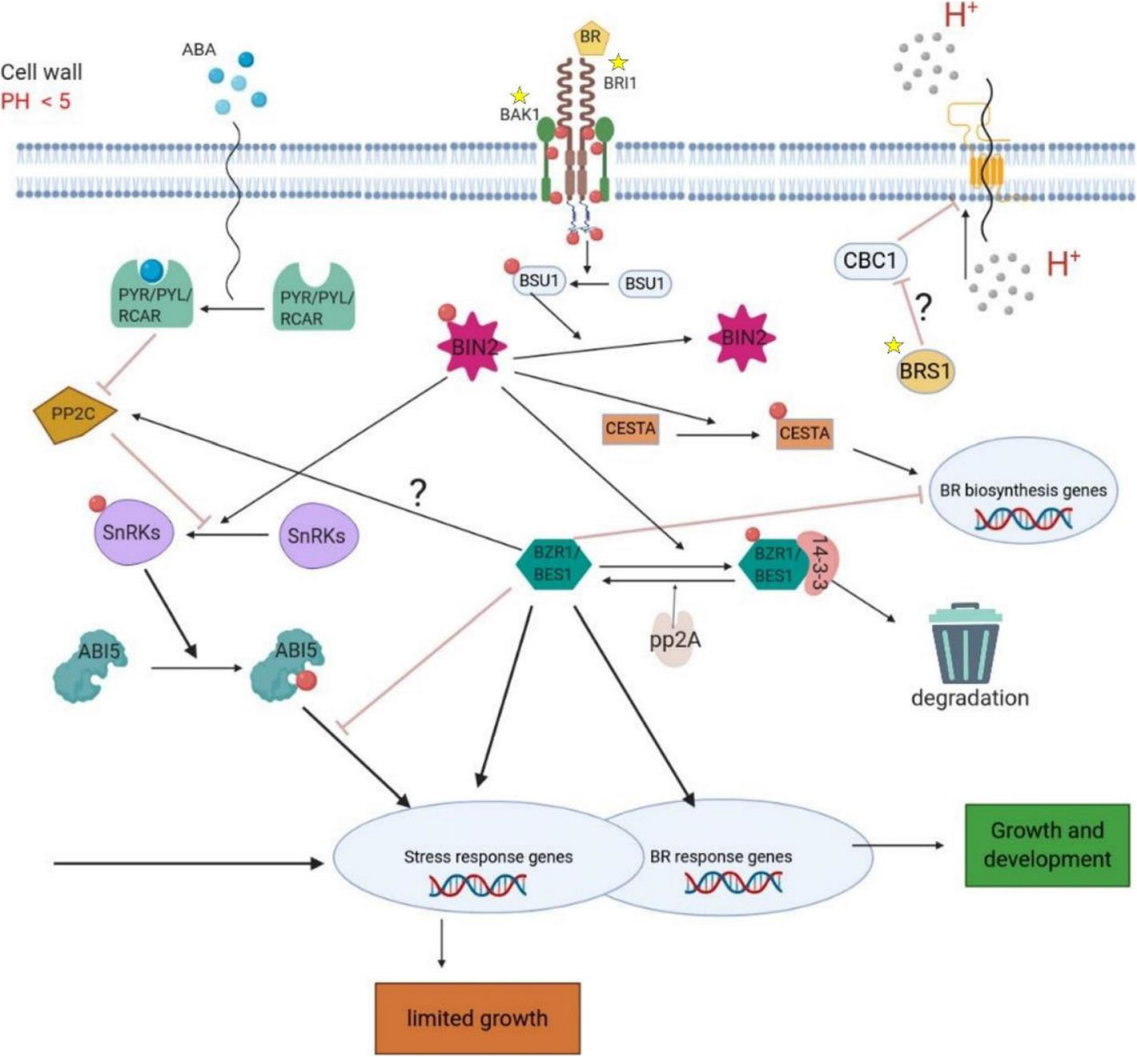
Schematic overview of the BR signaling cascade and the results of this study. The figure provides a simplified scheme of BR signaling based on [4, 5]. ‌The genes studied in this work are indicated by a yellow star. Binding of BRs to the BRI1/BAK1 receptor triggers the phosphorylation/dephosphorylation signaling cascade that leads to the deactivation (dephosphorylation) of BIN2. The effects of BIN2 and BZR1/BES1 on BR-biosynthesis genes are depicted. The overlap between stress-response and BR response genes and the dual effect of BZR1/BES1 on stress response genes is also shown. The question marks indicate missing links that have been suggested based on the result of the present study. The hypothetical inferred role for BRS1 in providing a better condition for BRI/BAK1/BR binding by generating a more acidic environment is shown on the top right-hand side. The compensatory pathway resulting in the over-expressing expression of PP2C mediated by ABA is shown on the left-hand side. (Created with BioRender.com)

Brassinosteroids (BRs) are essential plant hormones, regulating multiple processes amongst which plant growth, flowering, senescence, and seed germination [1]. BR biosynthetic and signaling mutants display aberrant morphological phenotypes such as dwarfism, reduced fertility, impaired photomorphogenesis, and altered vascular development [2, 3]. Whereas the phenotypes of mutants in BR biosynthetic genes can be rescued by the application of exogenous BRs, this is not the case for strains carrying mutations in genes responsible for BR signal perception and transduction. Hence these latter strains are referred to as BR insensitive (*bri*) mutants [1, 3]. In the BR signaling pathway (Fig. 1), BRs are perceived by membrane-localized leucine-rich-repeat-receptor kinase BRI1 or by the BRI1-like homologs, BRL1 and BRL3 [6, 7]. After binding to BRs, BRI1 and its co-receptor BRI1-Associated Receptor Kinase 1 (BAK1) phosphorylate each other. This results in triggering a cytoplasmic phosphorylation/dephosphorylation signaling cascade which deactivates the GSK3-like kinase BRASSINOSTEROID INSENSITIVE 2 (BIN2) through dephosphorylating [4, 5]. Upon BIN2 deactivation, the downstream transcription factors, BRASSINAZOLE-RESISTANT1 (BZR1) and BR-INSENSITIVE-EMS-SUPPRESSOR1 (BES1) are dephosphorylated by PP2A (PHOSPHATASE 2A). This results in their disassociation from 14-3-3 proteins, causing them to get activated and regulating a range of downstream genes involved in various aspects of plant growth and development [8–10]. In the absence of BRs, BIN2 is active (phosphorylated) and it prevents the activation of BZR1 and BES1*.* Because BRI1 is the core receptor of BRs, mutants of *BRI1* have been used as genetic background to identify suppressors, i.e. other genes that when mutated, suppress the *bri1* phenotype and thus may play a role in BR signaling. For example, the function of *BZR1* has been unveiled by using the null allele of *BRI, bri1–116* [11]. The weak mutant of *bri1*, *bri1–5*, can be rescued by overexpression of *BAK1* and *BRI* Suppressor 1 (*BRS1*) [3, 12]. BRS1 is a secreted member of the serine carboxypeptidase (SCP) family [3]. The fact that overexpression of *BRS1* can suppress two weak *BRI1* extracellular domain mutants, *bri1–5* and *bri1–9*, but not the strong cytoplasmic domain mutant *bri1–1*, implies that *BRS1*, unlike the downstream genes, *BZR1* or *BES1*, may function upstream of the BR signaling pathway or in a close regulatory relationship with *BRI1* [3]. Moreover, three of the five overexpressed *BRS1*’s homologs amongst which *ECS1* (Extra Carpels and Seeds 1) can also partially suppress the phenotype of the *bri1–5* mutant observed in leaves. Overexpression of *BRS1*’s homologs also increases the number of carpels and seeds, confirming the role of *BRS1* and its homologs in the BR signaling [13]. Yet, the detailed mechanism of how *BRS1* potentially interacts with other BR genes in order to maintain balance in BR signaling is still unknown.

Some genes involved in BR signaling are also involved in other processes, such as stress response, and can act independently of the presence of BRs. Several studies found that *bes1–1D* and *bzr1–1D* backgrounds are not responsive to exogenous BRs, suggesting that *BES1* and *BZRI* have also other functions than BR signaling [14, 15]. In another study, BAK1 was found to work together with Flagellin-Sensitive 2 (FLS2) during pathogen defense programmed cell death independently of BR signaling [14, 16–18]. In addition, SERK1 and SERK2, the homologs of BAK1 play a role in male microsporogenesis, also independently of BR signaling [19]. Some *bri1* mutants show in addition to reduced growth, an increased stress-tolerance, further confirming the complexity and dosage sensitivity of BR signaling and regulation [20, 21]. Transcriptomic studies and network analysis have shown to be effective in uncovering the expression and biological consequences of gene mutants, and have successfully been applied to study several BR genes such as *BRI1* and *BES1* [22]. Therefore, in the present study, we applied a similar strategy to elucidate the role of *BRI1*, *BAK1,* and *BRS1* in regulating/restoring the response to BRs and/or in other functions independent of BR signaling.

## Results

### *bri1–5/bak1–1D*, *bri1–5/brs1–1D* and *bri1–5/bri1–1D* partially reconstitute *bri1–5* gene expression

**Fig. 2:**
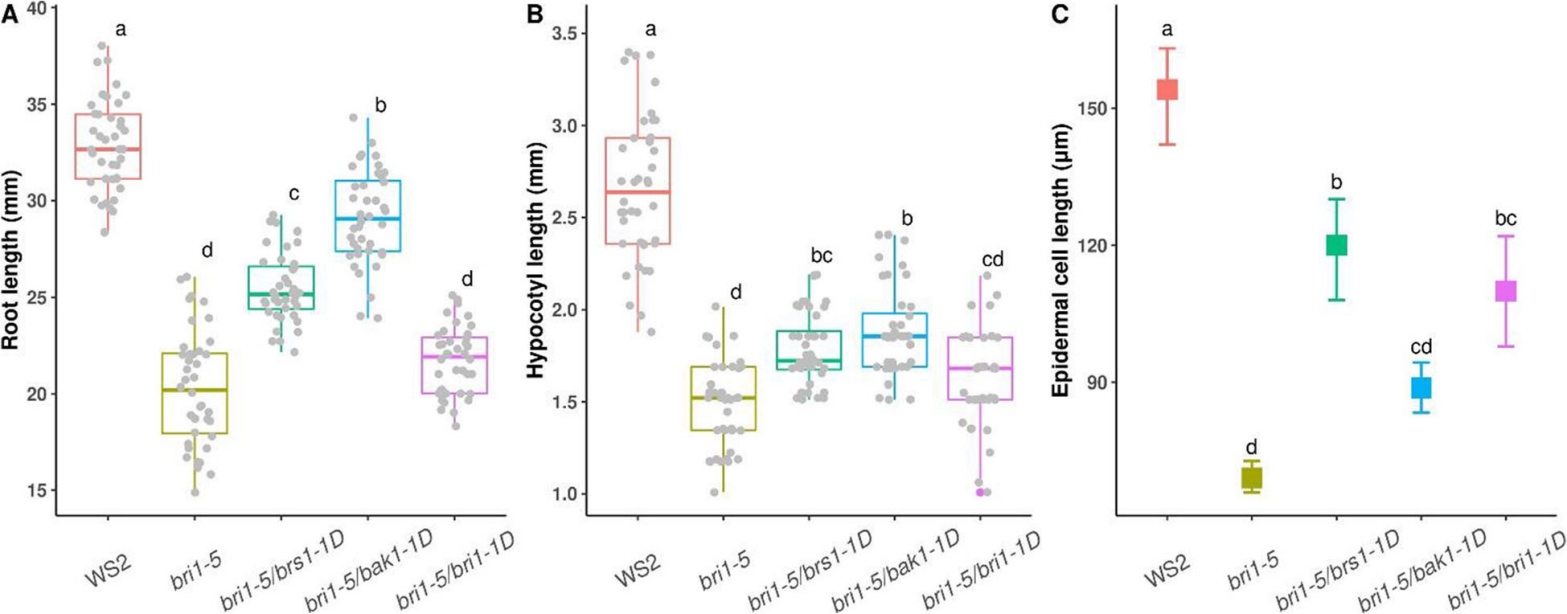
Root, hypocotyl, and epidermal cell length at seedling stage of plants used for expression profiling. Root (A), hypocotyl (B), and epidermal cell length (C) of WS2, *bri1–5*, *bri1–5/brs1–1D*, *bri1–5/bak1–1D,* and *bri1–5/bri1–1D*, measured 7 days after germination. For the root and hypocotyl length, the boxplot shows the distribution of data for 40 plants. For cell length, the bar represents the 95% confidence interval for the mean and the square indicates the location of the mean. Groups (different plant lines) were statistically compared by ANOVA and Tukey tests. Groups are ranked based on their significance level where “a” is representing the group with the highest mean and “d” the group with the lowest mean. Groups with different letters are significantly different

To better understand the molecular mechanisms of key BR signaling genes, we performed a phenotypic screening and expression analysis of *bri1–5* and its three activation-tag suppressors along with their corresponding wild-type, WS2. Two suppressor strains *bri1–5/bak1–1D* and *bri1–5/brs-1D* were obtained from [12]. An additional *bri1–5/bri1–1D* mutant was generated in the framework of the current study (see Methods). Sequencing the *BRI1* flanking region from the suppressor *bri1–5/bri1–1D* showed that the activation tag was inserted 534 bp downstream of the *BRI1* gene (Supplementary Fig. 1-A). All suppressor mutants were shown to indeed overexpress the activation tagged gene as confirmed by Real-Time qPCR (RT-qPCR) (Table S1). Phenotypically, all *bri1–5* suppressors (*bri1–5*/*bak1–1D, bri1–5*/*brs1–1D,* and *bri1–5*/*bri1–1D)* lines displayed larger seedlings than the *bri1–5* mutant, but still significantly smaller than the WS2 (Fig. 2). Of all suppressor mutants, the *bri1–5/bak1–1D* line best approximated the growth phenotype of the WS2, and its larger seedling seemed to be mainly the effect of its larger root length and to a lower extent of its larger hypocotyl length (both of which were significantly larger than the *bri1–5* mutant). The contribution of the epidermal cell length in recovering the *bri1–5* is marginal in the *bri1–5*/*bak1–1D* (line with the largest seeding) but seems much more pronounced in the *bri1–5/brs1–1D* (Fig. 2, Supplementary Fig. 1: B-F). This indicates that in the *bri1–5/brs1–1D* mechanisms other than those in the *bri1–5*/*bak1–1D* line play a role in alleviating the *bri1–5* phenotype.

To gain insight into which pathways in each of the studied lines were responsible for recovering the *bri1–5* growth phenotype to wild-type level, we performed gene expression analysis. All suppressor lines, together with the wild-type (WS2) and the *bri1–5* background were sampled at a 7-day seedling stage. To assess the reproducibility of the expression analysis, we measured the extent to which the expression profiles of replicate samples were similar using Principal Component Analysis (PCA): PCA indeed showed that the largest fraction of the variation in gene expression between the samples could be assigned to differences in genetic background and not to differences between replicates of the same genetic background, confirming the reproducibility (Fig. S2). In addition, microarray results were confirmed using RT-qPCR for a randomly selected set of differentially expressed genes (Fig. S3).

**Fig. 3:**
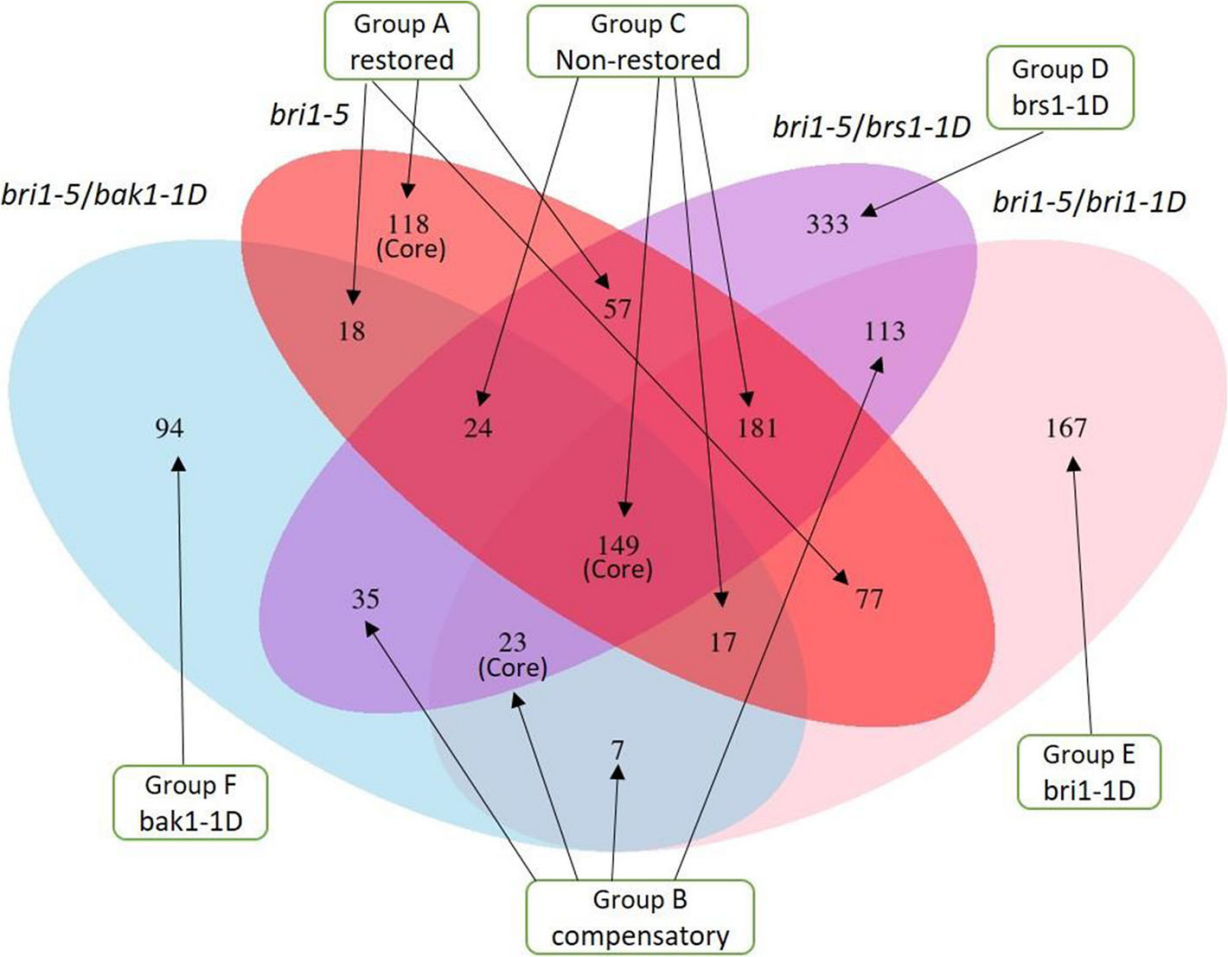
Differentially expressed genes (DEGs relative to WS2) being compared between *bri1–5* and its three suppressors. Group A (restored genes, 270 genes): genes differentially expressed in the *bri1–5* mutant but no longer in at least two of the suppressor lines; Group B (compensatory genes, 178 genes): genes that are differentially expressed in at least two suppressors but not in *bri1–5*; Group C (genes that were not restored, 371 genes): Genes that are aberrantly expressed in *bri1–5* and at least two of the suppressor lines. Group D (333 genes), E (167), and F (94 genes) contain genes that are exclusively differentially expressed in respectively the *bri1–5/brs1–1D*, *bri1–5/bri1–1D,* and *bri1–5/bak1–1D* suppressor lines. The “core” below the number indicates the most reliable set for the group. The total number of potentially interesting genes is 1430

We determined for each mutant line its differential expression versus the same common reference i.e. the expression state in WS2, resulting in a total of 1413 differentially expressed genes (Additional file 1). The Venn diagram represented in Fig. 3 shows to what extent the different lines share the same differentially expressed genes (aberrantly expressed versus the WS2 control). Fig. 3 and the scatter-plots in Fig. S4 (A-C) show that of all suppressor lines, *bri1–5/bak1–1D* could restore the largest number of genes that were affected in expression in *bri1–5* (about two-thirds of the genes that were differentially expressed in *bri1–5* were no longer differentially expressed in *bri1–5/bak1–1D*). This is in line with its observed phenotypic behavior as indeed *bri1–5/bak1–1D* seems to also phenotypically best compensate for the *bri1–5* mutation.

The Venn diagram in Fig. 3 also shows that the *bri1–5/brs1–1D* and *bri1–5/bri1–1D* lines share the largest fraction of similarly affected genes. The latter is also illustrated in Fig. S4 panel D-F which shows that from all pairwise comparisons between suppressor lines, the level of differential expression relative to the mutant *bri1–5* is most correlated between the suppressor lines *bri1–5/brs1–1D* and *bri1–5/bri1–1D* (i.e. *R*^2^ = 0.20). This suggests a similar role for *BRI1* and *BRS1* in the BR signaling pathway. Note that in Fig. S4 D-F, rather than performing a direct correlation analysis of the expression between two mutant lines, we performed correlation analysis with the expression of each mutant line relative to the same reference (expression in *bri1–5*). In this way, the correlation analysis is driven by the expression of the genes that change their expression relative to *bri1–5*. Although this results in lower correlation values than when directly comparing the expression values of the mutant lines, it better reflects the consistency between mutant lines in restoring genes affected in the *bri1–5* mutant.

**Fig. 4:**
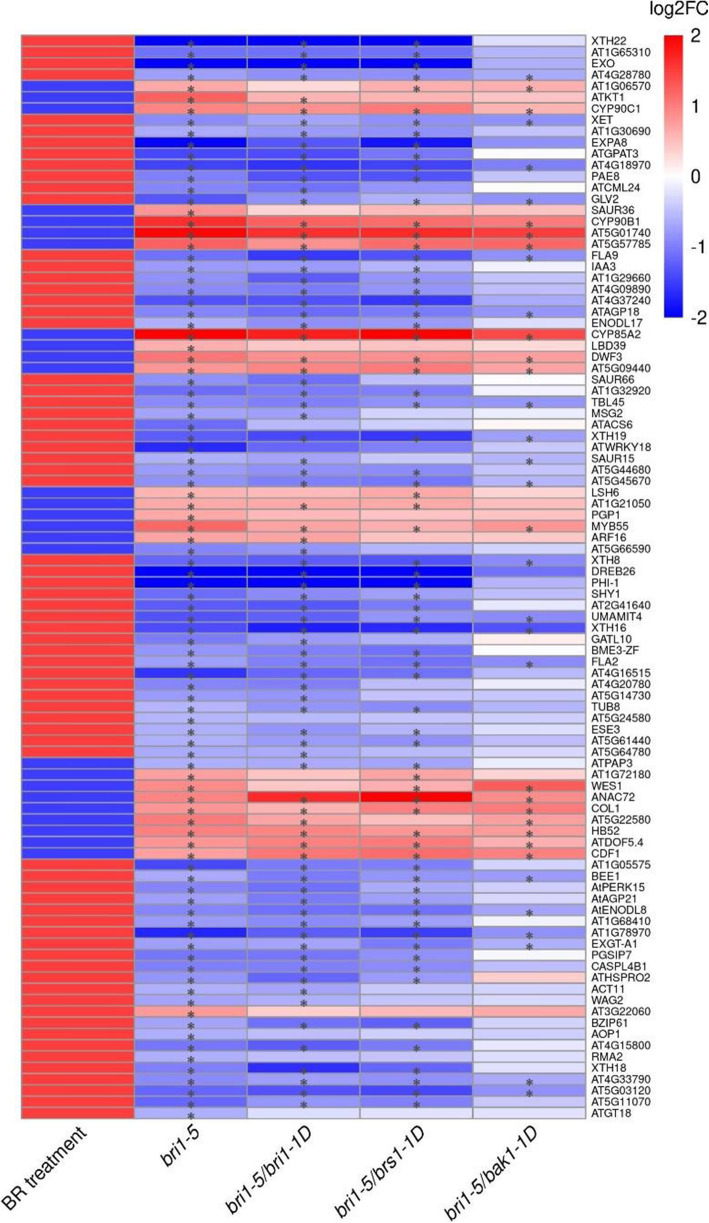
Expression behavior of marker genes representative of downstream BR signaling pathways. Column BR treatment: colors indicate whether a gene was reported to be up (red) or down (blue) regulated according to literature upon treatment with exogenous BRs or in a line containing a gain-of-function mutation in a BR signaling gene. Genes were only selected as representative for downstream BR signaling if the up/down regulation of their expression was confirmed by at least 5 independent references and also affected in the *bri1–5* line of our study (compared to WS2). Columns *bri1–5*, *bri1–5/bri1–1D*, *bri1–5/brs1–1D*, *bri1–5/bak1–1* indicate whether the genes were found to be up or down-regulated compared to WS2 according to our expression data. Color scale indicates whether a gene is up-regulated (red), down-regulated (blue), or not differentially expressed (white). A * indicates that the adjusted *p*-value < 0.05

To confirm the extent to which the different suppressor strains molecularly restore the defects in the *bri1–5* mutation, we compiled a list of marker genes representative of downstream pathways affected by BR signaling (Additional file 2). This consisted of 233 marker genes that were according to literature regulated by BR signaling (genes that became up or down-regulated upon treatment with exogenous BRs or by overexpressing the BR signaling genes). Of those marker genes, only those that were significantly affected in the *bri1*–5 line were retained in order to identify the mutant line that best suppresses the *bri1–5* mutation (96 marker genes). Fig. 4 and Fig. S5 show how the expression of these genes is, as compared to WS2 affected in the *bri1–5* mutant and how some of those genes got restored in the suppressor mutants. These results confirm what we observed based on the global expression analysis, i.e. that the *bri1–5/bak1–1D* restored the *bri1–5* affected marker genes to the largest extent, and that molecularly the *bri1–5/brs1–1D* and *bri1–5/bri1–1D* mutant tend to behave more similarly in restoring the same marker genes.

### Identifying compensatory and restoring pathways

Pathway analysis (see Methods) unveiled the pathways overrepresented amongst the differentially expressed gene sets in each of the mutant lines. Fig. S6, S7 and S8 and Table S2 show a number of pathways that are differentially expressed in both *bri1–5* and all of the suppressor lines. These represent the pathways that are responsible for the aberrant growth phenotype in the *bri1–5* mutant and that could not entirely be restored or compensated for in the suppressor lines. Among others, pathways related to cell wall synthesis (cell wall cellulose synthesis), protein and lipid metabolism can explain the residual discrepancy between the WS2 growth phenotype and the suppressors.

We assumed that if the suppressor strains alleviate the phenotype of the *bri1–5* mutant, they could do so because they either restore the pathways disrupted in the *bri1–5* mutant to wild-type levels or they induce genes that compensate for the *bri1–5* affected pathways. Both mechanisms are reflected in the expression data. Processes that are aberrantly expressed in the *bri1–5* mutant, but not in any of the suppressor lines, represent pathways that are restored to WS2 levels in all of the suppressors. This seems to be the case for some genes related to cytochrome P450 oxidase (Table S2). The fact that they are restored (or not significantly affected) in any of the suppressors indicates they might be essential for the recovery of the WS2 phenotype. Interestingly, the genes related to “glutathione S transferases” (Fig. S7, Table S2) are largely down-regulated in the *bri1–5* mutant, restored to normal in *bri1–5/bri1–1D* and *bri1–5/bak1–1D* and up-regulated as compared to WS2 levels in the *bri1–5/brs1–1D* suppressor, indicating that some overcompensation is needed for this pathway in the *bri1–5/brs1–1D* background in order to restore the *bri1–5* phenotype. In addition, ABA-related metabolism (Fig. S8 and Table S2) seems to have been affected by all suppressors, but at least not to a significant level in the *bri1–5* mutant. Therefore, ABA signaling seems to represent a compensatory pathway, i.e. a pathway that needs to be triggered in the suppressor strains in order to restore the *bri1–5* affected pathways and phenotype.

As less than 5000 genes can be mapped using pathway analysis, we performed a more elaborate analysis using a network-based approach. Network analysis provides an intuitive way of combining expression data with prior information on known molecular interactions or already available functional data [23, 24]. This approach first maps candidate genes, that are identified through expression analysis, on an integrated molecular interaction network. Then, it identifies subnetworks that connect as many candidate genes as possible [24]. By leveraging candidate genes identified through expression analysis with known interaction information, spuriously identified candidate genes can be removed as they will not be part of the subnetworks. In addition, genes relevant to the process of interest that are themselves not regulated at the level of expression are indirectly identified by being part of a connected component/subnetwork to which also many of the candidate genes belong. Such an integrated analysis provides a more comprehensive view of the process of interest. Here, we applied such an integrated network-based strategy to gain a more in-depth insight into the molecular mechanisms through which the suppressor lines can restore the *bri1–5* phenotype to WS2 levels. To perform this network analysis, we started from the gene sets depicted in Fig. 3.

### Involvement of hormone signaling in alleviating the *bri1–5* phenotype

To study the interaction between non-restored, restored, and compensatory pathways in more depth, we combined the following gene sets for network analysis (see Fig. 3): i) genes that were most likely restored in the suppressors (genes of group A i.e. the genes with altered expression in the *bri1–5* mutant, but restored to WS2 level in at least 2 suppressors), ii) genes that were compensatory in most of the suppressors (genes of group B i.e. the genes not differentially expressed in the *bri1–5* mutant, but differentially expressed in at least two suppressor strains) and iii) genes altered in the *bri1–5* mutant that most likely were not restored in the suppressors (genes of group C i.e. the genes, differentially expressed in the *bri1–5* mutant and at least two of the suppressor strains). This combined set of genes (789 genes) is referred to as the set of seed genes or the genes we want to maximally connect on the interaction network.

**Fig. 5:**
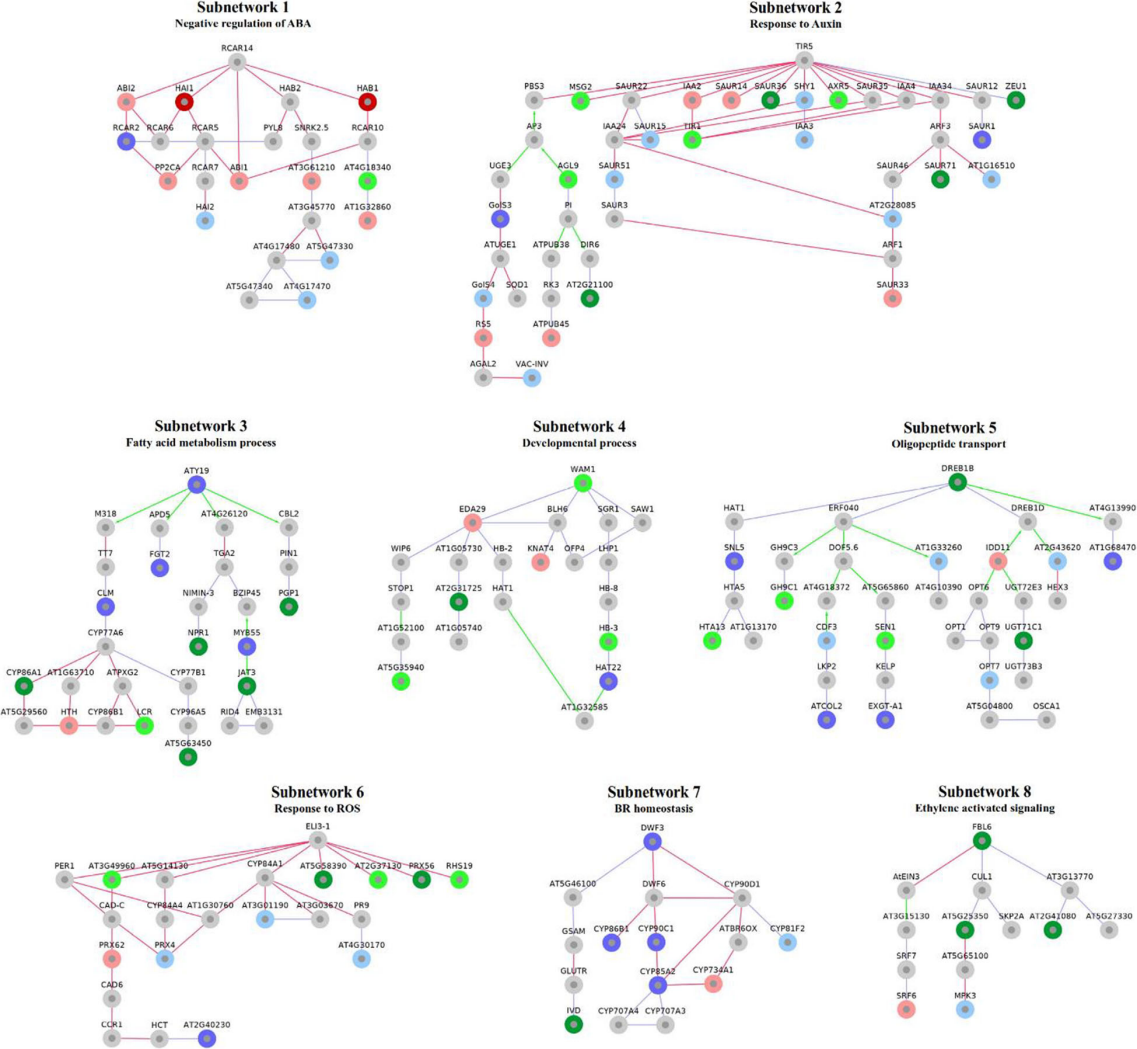
Subnetworks resulting from network analysis. Subnetworks identified by PheNetic representing different pathways that were identified by mapping and connecting the genes of group A (restored genes), B (compensatory genes), and C (not restored genes) on the interaction network. Node color: dark green, dark red, and dark blue indicate the core genes of groups A, B, and C, respectively. Likewise, the light green, light red, and light blue correspond to the non-core genes in groups A, B and C, respectively. Connector genes that were not identified as differentially expressed, but identified by PheNetic on the paths that connect the differentially expressed genes are shown in gray. Edge color: regulatory edges are shown in green, metabolic edges in red and protein-protein edges are shown in blue color

Network analysis (see Material and Methods) identified 8 sub-networks (Fig. 5) containing the set of seed genes that could be connected through the interaction network. These subnetworks contain not only seed genes, but also connector genes. These are genes that are not differentially expressed themselves, but that are still recovered by the network analysis, because of their high connectivity with seed genes. As they are needed to connect seed genes in the network, they are most likely involved in the same processes as the seed genes. The subnetworks were annotated based on their enrichment in known GO functions (being enriched in respectively negative regulation of ABA, response to auxin, fatty acid metabolism process, developmental process, oligopeptide transport, response to ROS, BR homeostasis, and Ethylene activated signaling (Fig. 5)). This indicates that these are the pathways that contribute to alleviating *bri1–5* signaling deficiency in the suppressor strains.

In-depth analysis shows that the subnetwork enriched in ABA signaling (Fig. 5, subnetwork 1) contains several known negative regulators of ABA signaling: *HAI1*, *HAB1 ABI1*, *ABI2*, and *PP2CA* acted as compensatory genes: these were are up-regulated in at least two *bri1–5* suppressor lines compared to wild-type, but were not affected in the *bri1–5* mutant (*HAI1* and *HAB1* being significantly up-regulated in all suppressor mutants; *ABI1*, *ABI2*, *PP2CA*, being significantly up-regulated in two suppressors, see Fig. S9); In addition, *HAI2* was affected in the *bri1–5* line, but could not be restored in at least two suppressors (non-restored gene), and *HAB2* was identified as a connector node.

Interestingly, several targets of the ABA signaling pathway (*DTX50, HVA22D, PUB19, COR15B, next to HAI1, HAB1)* were identified as differentially expressed in all three suppressors (identified based on a GO enrichment of the core of group B, but not in *bri1–5*). This indicates that ABA signaling has indeed been affected in the suppressor strains to compensate for the *bri1–5* signaling deficiency. Of these, *DTX50, HVA22D, PUB19, COR15B* could not be connected by PheNetic on the interaction network, implying they are either not annotated in the interaction network (*COR15B*) or quite distantly located from each other in the network.

The aforementioned negative regulators of ABA signaling in subnetwork 1 belong to the protein phosphatase 2C (PP2C) gene family which has nine members in total (*HAI2*, *HAB2*, *HAB1*, *HAI3*, *PP2CA*, *ABI1*, *AHG1*, *ABI2*, *AHI1*). PP2C is known to indirectly repress ABI5, the main activator of ABA signaling [25]. PP2C is also known to repress BIN2 activity [4, 5]: as BIN2 activates ABI5 by phosphorylating SnRKs [4, 5], repressing ABA signaling by PP2C via blocking SnRKs phosphorylation seems to compensate for the deficiency in BRI1 mediated signaling (Fig. 1). The subnetwork enriched in ABA signaling (network 1) also contains members of the *PYR/PYL/RCAR* family as connector genes (*RCAR5*, *RCAR6*, *RCAR7*, *RCAR10*, *RCAR14*, *PYL8*). The PYR/PYL/RCAR family constitutes the receptor of ABA signaling and promotes the activation of SnRKs by repressing PP2C [15, 26]. The fact that the SnRKs (SnRK2.5) and PYR/PYL/RCAR genes were identified as connector genes implies that they are likely involved in the pathways that connect the affected, restored, and compensatory genes of subnetwork 1. They are most likely not primarily regulated at the expression level, given their role in phosphorylation-mediated signaling [5, 27]. This explains why they were detected as connector genes and not retrieved by differential expression analysis.

We could not find any link in the literature to explain how *PP2C* can be up-regulated by BR signaling in order to repress ABA signaling. It seems that there exist some missing links between *BZR1/BES1* (or downstream TFs) and the *PP2C* gene family. By partially recovering BR signaling in the suppressor lines, one would expect that *PP2C* gene expression levels restore to WS2 expression level. However, they appear to become up-regulated in suppressors, indicating that further compensatory repression of the ABA signaling is required in order to restore the *bri1–5* phenotype.

Other than the ABA subnetwork, subnetworks related to other hormone signaling processes like auxin signaling (subnetwork 2), ROS signaling (subnetwork 6), and ethylene signaling (subnetwork 8) were also detected. It is well known that crosstalk between these phytohormone signaling pathways exists [28, 29]. Hence, interfering with one pathway e.g. *ABA* signaling through BR signaling might affect other phytohormone pathways as well. Based on the pathway analysis using MapMan and the network analysis we can conclude that ABA signaling is mostly a compensatory pathway (genes indicated in red color in Fig. 5 represent compensatory genes), whereas auxin, ethylene, and ROS signaling pathways are at least partially restored to the WS2 level (genes indicated in green color in Fig. 5 represent restored genes). However, restoring those pathways to the WS2 level seems to also depend on the presence of at least some compensatory genes (genes indicated in red color in Fig. 5 represent compensatory genes). The remaining subnetworks are enriched for fatty acid metabolism (subnetwork 3), developmental processes (subnetwork 4), and oligopeptide transport (subnetwork 5) confirming that all suppressors could partially restore some affected primary metabolic pathways. This is in line with the MapMan results and the recovered phenotypes.

### Negative feedback of BR signaling on BR biosynthesis

**Fig. 6:**
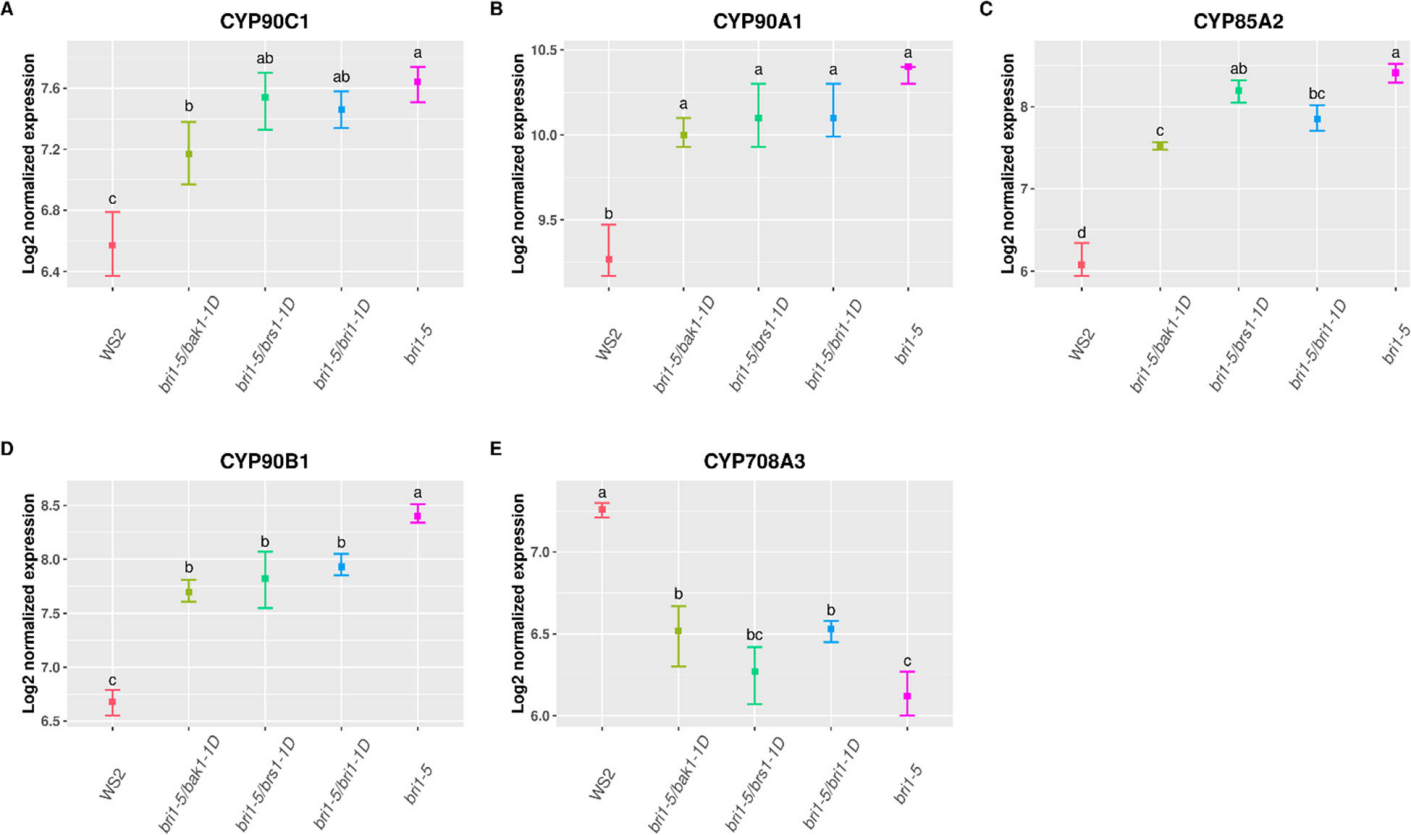
Comparing the mean expression values of BR-biosynthesis genes in the *bri1–5* mutant and suppressor lines. For each line (WS2, *bri1–5,* and suppressors) the average log2 expression values of gene expression across replicates are given for the indicated BRs biosynthesis genes. The squares indicate the location of the mean and bars show the 95% confidence interval for the mean. The main BR-biosynthesis genes are affected in the *bri1–5* mutant and all suppressors. However, the plots show that BR-biosynthesis genes are less affected in the line (*bri1–5/bak1–1D)* that best suppresses the *bri1–5* phenotype*.* Pairwise comparisons between the average values were performed using Tukey’s post hoc test. Groups are ranked based on their significance mean where “a” is representing the group with the highest mean and “d” the group with the lowest mean. Groups indicated with different letters are significantly different

The network result shows that the BR biosynthesis (subnetwork 7) is not well recovered in any of the suppressors. This subnetwork contains 4 genes (*CYP90C1*, *CYP90B1/DWF4, CYP85A2, CYP90A1/DWF3)* that are differentially expressed in the *bri1–5* lines and all of the suppressors. Those genes, belonging to the cytochrome P450 superfamily play a role in BR biosynthesis by converting the sterol “campesterol” to BRs [30]. *CYP708A3*, another BR biosynthesis gene was found to be differentially expressed in all suppressors and the *bri1–5* mutant (Fig. 6). However, as this gene was not present in the interaction network, it was missed by the network analysis. Unlike the four aforementioned cytochrome P450 genes, the molecular function of the *CYP708A3* gene is still unknown. Interestingly it shows an expression pattern that is anticorrelated to that of the other cytochrome P450 genes (Fig. 6). This result along with the fact that *CYP708A3* is known to be up-regulated by exogenous brassinolide (BL) treatment [31, 32] suggests it acts as an inhibitor of BRs biosynthesis*.*

The fact that the expression of BR biosynthetic genes is affected by mutations in BR signaling genes points towards the existence of negative feedback of BR signaling on BR biosynthesis. If indeed negative feedback exists between BR signaling and biosynthesis, this feedback should be reflected in quantitative differences in overexpression of the BR signaling and biosynthesis genes in the *bri1–5* and suppressor mutants. The better the signaling can be restored in the suppressors (as reflected by the phenotype), the less we expect the expression of the BR biosynthesis to be aberrant. We indeed found that the expression of the BR-biosynthesis genes (*CYP90C1*, *CYP90A1*, *CYP85A2*, *CYP90B1, CYP708A3*) are less affected in the strains that better mimic the wild-type phenotype (see Fig. 6, the best suppressor of *bri1–5*, *bri1–5*/*bak1-D,* shows the lowest expression change of the biosynthesis genes). This further supports the existence of negative feedback from BR regulation on BR biosynthesis: a more sustained BR signaling results in decreased BR biosynthesis, whereas suboptimal BR signaling is compensated for by higher transcriptional activity of BR biosynthetic genes.

### Link between stress response and BR signaling

Next, we analyzed the genes that were uniquely altered in each of the suppressors, i.e. the suppressor-specific compensatory genes (genes of group D, group E, and group F, respectively). Based on GO analysis we mainly found stress related processes to be overrepresented in each of the groups. Genes involved in these stress related processes seem to be scattered over the interaction network as they could not be recovered as well delineated subnetworks, indicating that different stress-related genes are induced in the different lines. According to the literature, there is crosstalk between BR signaling and signaling by other hormones in response to stress, especially via ABA and auxin signaling [28]. In the absence of BRs (or low amounts of BRs), BIN2, affects ABA and auxin signaling, resulting in the induction of stress response genes [4, 5, 15]. On the other hand, some stress-response genes are known to be targets of *BZR1* and *BES1* [5, 15] (Fig. 1), indicating that also when BRs levels are high, stress response genes can be activated. These observations show that balanced BRs levels are needed for normal growth and that deviation from the optimal levels (either too high or too low) would activate stress response mechanisms. We observed that by partially recovering *bri1–5* signaling deficiency by suppressors mutants, the transcript level of some stress-response genes is restored to normal, but other stress-response genes become induced (Additional file 3: GO enrichment for genes exclusively differentially expressed in each suppressor, “GO_only_bri1–5”, “GO_only_bri1–1D”, “GO_only_bak1–1D”, “GO_only_brs1–1D”). This observation is in line with this complex effect of BRs and BR signaling on stress response pathways.

### Iron ion homeostasis, ferroxidase, and glutathione transferase activity are identified as compensatory mechanisms unique to the *bri1–5/brs1–1D* suppressor

**Fig. 7:**
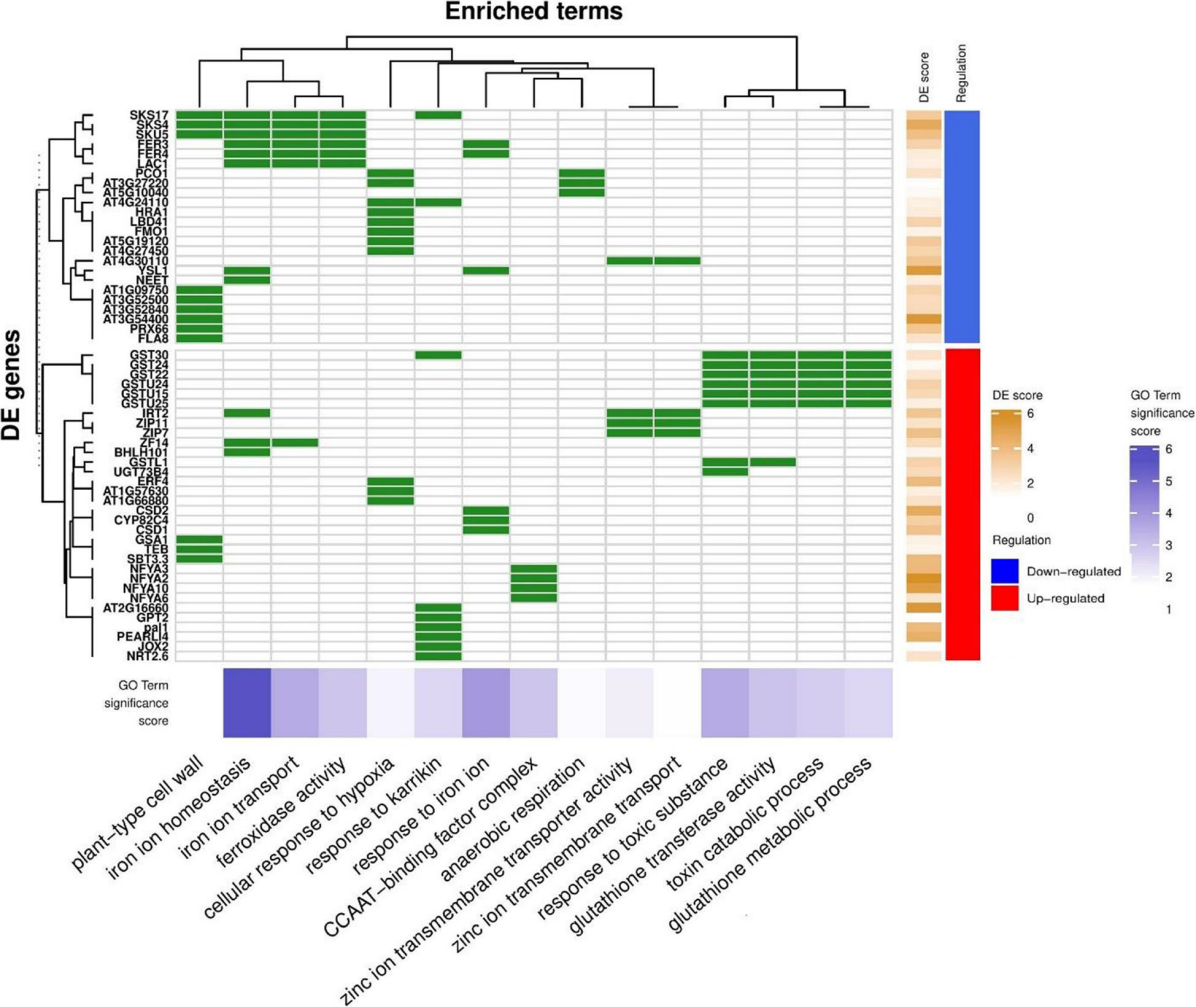
GO enrichment for differentially expressed genes (DEGs) exclusively in bri1–5/brs1–1D compared to WS2. The over-represented GO terms and DEG are shown on the x-axis and left-side y-axis, respectively. The green color shows the corresponding gene is present in the indicated GO term and white means it is not; “DE score” reflects the degree of log fold changes (differential expression compare to WS2); “Regulation” represents down (blue) and up (red) regulation for the corresponding gene. The small bottom heat map shows the significant over-representation value for GO terms based on the p-value in the hypergeometric test.

Unlike for BRI1 and BAK1, much less is known about the role of BRS1 in BR signaling. Therefore we had a closer look at genes of group D which are exclusively differentially expressed in *bri1–5/brs1–1D* mutant and hence comprise compensatory pathways specific for *bri1–5/brs1–1D*. GO enrichment showed that the genes of this group (group D, 333 genes) are not only overrepresented in stress related processes (see above) but also in glutathione transferase (up-regulated), (Fig. 7). This overrepresentation in glutathione transferase is in line with the MapMan results. These results showed how in the *bri1–5/brs1–1D* mutant the expression of the glutathione transferase was not only restored as compared to the other suppressor lines, but even overcompensated as compared to WS2 levels (Fig. S7). We also found that several members of the CCAAT-binding factor complex (CBC) (*NFYA2*, *NFYA3*, *NFYA6*, *NFYA10*) were uniquely up-regulated in *bri1–1D/brs1–1D* (Fig. 7 and Fig. S9). Members of this complex have been associated with the control of iron homeostasis in *Candida glabrata* [33]. In addition, iron ion homeostasis/ferroxidase activity was also found to be down-regulated, specifically in the *bri1–5/brs1–1D*. In the ferroxidase reaction, four H^+^ are used to catalyzes the oxidization of Fe^2+^ to Fe^3+^, repressing this reaction results in the accumulation of H^+^ which can be transported to the apoplast via plasma-membrane pumping mediated by ATPase (H + -ATPase transporters) [34]. Accordingly, we also found that the main inhibitor of H^+^-ATPase transporters, *CBC1*, was significantly down-regulated in *bri1–5/brs1–1D (*fold change − 1.7, adj *p*-value 8.36e-06), but not in the other suppressors. This implies that H^+^-ATPase transporters are more active in *bri1–5/brs1–1D* to export H^+^ from cytosol into apoplast, making the apoplast more acidic (Fig. 1). In line with this hypothesis, the up-regulated glutathione transferase activity in the *brs1–1D* mutant (Fig. S9) might be essential to compensate for the more acidic environment in the *bri1–5/brs1–1D* and would be required for maintaining redox homeostasis. In addition, we hypothesize that the observed acidification could generate a cellular environment that improves BRI1-BRs binding or BRI1-BAK1 dimerization and hence contributes to restore the *bri1–5* mutant phenotype.

## Discussion

In this study, we explored the alteration of gene expression in BR signaling mutants to better understand BR signaling and the functions of key BR genes.

### Crosstalk between BR signaling, other hormone signaling pathways, and primary metabolites

Our analysis identified ABA signaling as a mainly compensatory pathway, and hormone signaling pathways related to ethylene, auxin, and ROS, as pathways involved in partially restoring the *bri1–5* expression phenotype to WS2 levels. It is also obvious that restoring pathways can depend on the presence of compensatory genes in the same pathway. The crosstalk between BR signaling and signaling by ABA observed in our study is in line with the literature [35]. The serine-threonine kinases SnRK2.2 is the main positive regulator of ABA signaling by regulating key TFs such as *SLAC1*, *KAT1* [36, 37]. PHOSPHATASE 2C (PP2C) represses the SnRK2.2 by blocking its phosphorylation mediated by BIN2 (Fig. 1). In the presence of ABA, the complex of PYR/PYL/RCAR inactivates the PP2C by blocking its substrate’s entry [38]. The activated SnRK2 phosphorylate ABI5 leading to the activation of downstream ABA-dependent mechanisms. In the absence of BRs (or BR signaling deficiency like *bri1–5*), SnRK2.3 can mimic the presence of ABA in triggering ABA signaling, once it is phosphorylated by BIN2 [25]. This means that repressing ABA signaling (repressing SnRK2) can compensate for the BR signaling deficiency. In line with our result, a recent study showed that overexpression of *ABI1* or *ABI2,* which encodes the negative regulator of ABA signaling could promote BR signaling [35]. Network analysis suggested that all *bri1–5* suppressor strains suppressed the ABA signaling (SnRKs) through up-regulating its negative regulator, *PP2C* (Fig. 1).

Links between BR signaling and auxin we observed are also supported by literature and in line with the observed phenotype of the *bri1–5* mutant. ARFs (auxin response factors) are transcription factors that affect root and shoot elongation [29]. In the presence of BRs, BZR1 and BES1 enhance the DNA-binding activity of the auxin response factors ARF6 and ARF7 to promote auxin response [39, 40]. This explains why the suppressor strains restore auxin-related pathways. However, it remains unclear whether the observed restored auxin signaling was the result of the activation of BZR1/BES1 or whether the suppressors employed other pathways to restore the auxin signaling. On the other hand, at low concentrations of BRs, BIN2 enhances the DNA-binding activity of the auxin response factors ARF2, ARF7, and ARF19 through phosphorylation. This results in growth and root elongation in the absence of BRs [37, 41]. Hence, optimal plant growth and development regulated by auxin signaling requires a balanced level of BR signaling. Like with ABA and auxin, crosstalk between BRs and respectively ethylene and cytokinin has also been reported [28].

BR signaling controls development and growth by regulating metabolic processes such as fatty acid metabolism. For example, exogenous BR treatment was shown to promote leaf senescence, likely via the alteration of fatty acid composition in *Pisum sativum* [42]. Exogenous BR treatment would increase the content of free fatty acids and decrease the content of fatty acids bound to galactolipids [42]. Similar studies showed the effect of exogenous BR on fatty acid composition under salt [43] and drought [44] stress. As expected, fatty acid metabolism (subnetwork 3) and developmental processes (subnetwork 4) have been partially restored by *bri1–5* suppressor lines, supporting the impact of BR signaling on the development, and the composition of fatty acids.

Transcription factors, regulators, oligopeptides, and proteins are an essential part of BR- and other signaling pathways. Previous studies showed that BR treatment increases the protein levels in the nuclei of hypocotyl cells [11, 45]. Transport of transcription factors and regulators to the nucleus is essential to regulate gene expression by BR signaling as a downstream effect [46]. This is in line with our results: oligopeptide transport (subnetwork 5) is affected in the *bri1–5* mutant and is partially restored by suppressor lines.

### Negative feedback between BR signaling and BR biosynthesis

In addition, our results provide evidence for negative feedback between BR signaling and BR biosynthesis. This hypothesis was already made by Noguchi et al. [2] who explained the observed accumulation of BR precursors in *bri1* mutants by the presence of a negative effect of the BR signaling proteins BZR1/BES1 on the BR-biosynthesis pathway. Although it cannot be excluded that the previously reported accumulation of BR precursors in *bri1–5* mutants results from the inability of the mutant lines to use available BRs, our observation in BR signaling mutants suggests that aberrant regulation of BR biosynthesis can also contribute to the accumulation of BRs precursors: it seems that non-aberrant BR signaling is required for homeostasis of appropriate levels of endogenous BRs. Our results also show that the level of negative feedback depends on the degree to which the suppressor could compensate for the phenotypic difference between the *bri1–5* and WS2. The better the defects in BR signaling were alleviated (as reflected by the phenotype), the less pronounced the observed effects on the BR biosynthetic genes. This observation also supports the hypothesis made by Gruszka et al. [4] that BIN2 would regulate BR-biosynthesis through phosphorylating CESTA, a transcription factor that regulates BR-biosynthesis (Fig. 1).

### BR signaling and stress response

Because BR signaling regulates response to a wide spectrum of stresses [15], it is not unexpected we observed that stress response genes were affected in the mutants that interfered with BR signaling. At the low level of BRs (or BR signaling deficiency e.g. *bri1–5*) the activated SnRK2 by BIN2 mimics the presence of ABA, activates ABI5, and finally regulates stress response genes (Fig. 1). In the presence of BRs, BZR1/BES1 inhibits ABI5 and can terminate the ABA signaling. On the other hand, BZR1/BES1 can regulate the expression of stress response independent of ABA (Fig. 1). Therefore, some stress response genes are regulated by BIN2 at low levels of BRs through ABA signaling, while other stresses are controlled by BZR1/BES1 at high levels of BRs independent of ABA signaling. We observed that by partially recovering *bri1–5* signaling deficiency in *bri1–5* suppressor strains, the transcript level of some stress-response genes is restored to normal, but other stress-response genes become induced. The need for optimal BR homeostasis might also explain why some gain-of-function mutants (e.g. *bes1-D*) described in the literature or why treatment with exogenous BR give rises to a phenotypic response that is worse than the one observed in the *bri1–5* mutant (shorter root) [47]. This further confirms that an appropriate balance in BR signaling is essential to guarantee coherent cross-talk between hormones signaling networks and any aberration of this optimal level leads to activation of stress response genes.

In addition, there is evidence that BAK1 plays a role in regulating stress-response pathways independently from BR signaling [16]. Since BAK1 usually works as a coreceptor and serves to promote cross-phosphorylation leading to downstream signaling, the existence of other stress-sensor receptors interacting with BAK1 cannot be excluded.

### Acidification possibly involved in providing an optimal environment for BRI1 and ligand binding

Our analysis of the genes/pathways that are uniquely involved in the *bri1–5/brs1–1D* suppressor line to compensate for the *bri1–5* mutant showed that BRS1 seems involved in the acidification of the apoplast environment. We hypothesize that this acidification could contribute to an improved BRI1-BRs binding or BRI1-BAK1 dimerization and hence restoration of the *bri1–5* mutant phenotype. In vitro studies have indeed shown that BRs preferentially bind to BRI1 in an acidic cell wall environment (pH < 5) [26, 48]. It has also been suggested that changing the pH environment by endocytosis of BRI1 from the plasma membrane into the cytosol reduces the affinity of BRI1 to BRs and would terminate BR signaling [26]. The same ligand-receptor mechanism has been reported in animal cells [49]. In addition, acidification of the apoplast is the major requirement for increasing cell wall extensibility, which controls cell extension and can also be a compensatory pathway in *brs1–1D* [50]. This is confirmed by the phenotypic analysis which shows that indeed the *bri1–5/brs1–1D* line at least partially restores the epidermal cell length (Fig. 2). These observations support that the *brs1–1D* mutant can restore BR signaling by creating an acidic environment and providing the optimal conditions for either BRI1-ligand binding or BRI1-BAK1 dimerization along with improving cell wall extensibility. This might also explain the very similar genome-wide expression impact of *brs1–1D* and *bri1–1D* and explains why overexpression of *BRS1* can suppress two weak BRI1 extracellular domain mutants, *bri1–5* and *bri1–9*, but not the strong cytoplasmic domain mutant *bri1–1.*

The *bri1–5/brs1–1D* suppressor line also induces glutathione transferase activity which is necessary for redox homeostasis. This link between BR and redox signaling is in line with the literature [51]. An oxidative environment induces BZR1 activity and promotes the interaction of BZR1 with downstream TFs, ARF6, and PIF4 [51]. Since a loss-of-function mutant of BRS1 shows no obvious phenotype, [3] but its gain-of-function mutant does and partially restores *bri1–5* signal deficiency, it can be suggested that BRS1 does not have a regulatory role and only provides a better condition for triggering the BR signaling by making the apoplast environment more acidic.

## Conclusions

In this study, we performed expression, pathway, and network analysis to provide more insight into the BR signaling by taking advantage of the availability of mutants for keys genes in BR signaling. Our results suggest that ABA signaling plays a significant role in alleviating the *bri1–5* dwarf phenotype. The fact that also other phytohormone signaling pathways are restored to the wild-type expression level in all *bri1–5* suppressors confirms the crosstalk between BR and other phytohormone signalings. The negative feedback from BR signaling on BR biosynthesis was also confirmed by quantitative evidence. In addition, a new indirect role for *BRS1* in BR signaling was suggested. However, our study is limited to transcriptome analysis and BR signaling is likely regulated to a large extent at the post-transcriptional level (i.e. phosphorylation). By using network analysis we can partially deal with this missing information, but this approach is limited by the incompleteness of the interaction network. Hence, more extensive validation studies are required to confirm our hypotheses. Besides, this study mainly focuses on the suppressor lines of *bri1–5*. Future work by adding enhancer lines such as *bri1–5/bri1–1*, *bri1–5/brs1–1*, *and bri1–5/bak1–1* or by studying the effect of suppressors not only in the *BRI1* as a loss-of-function background but also using double or triple mutants of multiple genes of the *BRI1* gene family and/or *BRI1*-like genes [52] could be interesting to further elucidate the mechanism of *BRI* signaling. In addition, future research by scRNA-seq of *bri1–5* suppressor mutants across different time points of hypocotyl growth would be key to elucidating the detailed mechanisms of BR signaling at the level of cell types [53, 54].

## Methods

### Expression profiling experiment and differential expression analysis

The two activation tagging suppressors of *bri1–5, bri1–5/bak1–1D,* and *bri1–5/brs1–1D* were obtained from our previous study [12]. An additional activation tagging suppressor line *bri1–5*/*bri1–1D* was generated in this study as previously described [3]. Wild-type (WS2), the loss-of-function BR mutant (*bri1–5*), and its three suppressor mutants (*bri1–5*/*brs1–1D*, *bri1–5*/*bak1–1D*, *bri1–5*/*bri1–1D)* were grown at 22 °C in a long-day condition (16 h of light and 8 h of dark) in a greenhouse for 7 days. All mutants were generated from the WS2 ecotype background. Microarray analysis of all genotypes was performed with three biological replicates. Total RNA of 7-day whole seedlings was isolated, labeled, and hybridized with an Arabidopsis ATH1 genome array according to the Affymetrix instructions. Scanning of the array was performed using the Agilent GeneArray Scanner. The data are available in GEO (GSE70843). The CEL files were preprocessed using the AFFY package (background correction, quantile normalization, and probe value summarization (RMA normalization)) [55]. The arrayQualityMetrics package was used to check the quality of the normalized expression values [56]. All samples passed the quality check. Non-unique probe sets were removed, and the expression value of genes was calculated as the average of the expression measured by the probe sets that covered the gene. The consistency between replicate samples was assessed using PCA. Differential expression was calculated by comparing the samples of the mutated lines with those obtained from the wild-type (WS2) using the Limma package [57]. To define differentially expressed genes, the absolute fold change and false discovery rate (FDR) threshold were set at 1.5 and 0.05, respectively resulting in a total of 1413 differentially expressed genes. Further, eight differentially expressed genes were randomly selected and confirmed by RT-qPCR following standard protocol with three biological and three technical repeats [58]. The primers used for qPCR are presented in Table S3. Pathway enrichment analysis was carried out using the MapMan software [59] and GO enrichment was performed using TAIR GO enrichment [60].

### Phenotypic analysis

The phenotypic impact of suppressor lines was evaluated through the measurement of the root, hypocotyl, and epidermal cell length on the 7-day old seedlings. The root and hypocotyl lengths were measured on 40 plants and hypocotyl epidermal cells were scanned using the electron microscope (SEM) on samples taken from 3 random plants for each line. The mean of epidermal cell length per image was determined using Fiji software [61]. Statistical analysis was performed using One-way ANOVA followed by Tukey’s multiple comparisons in R [62].

### Retrieving BR-responsive genes

We performed a literature study to compile a list of “high-confidence” BR-responsive genes, i.e. genes regulated by exogenous BR treatment or in a line containing a gain-of-function mutation in a BR signaling gene with consistent transcriptional response from at least five references [31, 32, 63–71]. A full list of marker genes together with the references in which they were found and an indication of their expression behavior upon addition of external BRs (or gain of mutation in BR signaling genes) is given in supplementary file (Additional file 2). The intersection of the genes that were differentially expressed in the *bri1–5* mutant line with the list of “high-confidence” BR-responsive genes was used to evaluate the consistency between the expression data of our study and literature and to determine which suppressor best restored the expression of those genes to WS2 level.

### Network analysis

A high confidence Arabidopsis interaction network was compiled from the following sources: 64185 FunTFBS regulatory interactions were obtained from PlantRegMap [57], 96,827 protein-protein interactions from AtPIN [72] and 34,003 metabolic interactions from KEGG [73]. This resulted in a final number of 182,748 interactions between 21,263 unique genes. In this integrated network, nodes represented genes and edges the interactions between the genes.

To perform network analysis with PheNetic [24] network edges need to be weighted. The weight is derived from the log2 fold change (logFC) expression of a gene as indicated below. To each gene, we assigned as logFC, the highest value that was observed for this gene across the assessed mutant lines as compared to WS2. To assign a *p*-value to this logFC, we empirically estimated the distribution of the observed max logFC for all genes. As we expected that most of the genes would not change their expression this is an estimate of the null distribution. The mean (μ) and standard deviation (σ) of this distribution was estimated empirically using maximum likelihood implemented in the MASS package [74]. Using the values of the mean and the standard deviation each gene was assigned a significance score (gene-score) based on a two-tailed T-test which reflects the degree to which the gene has been affected as compared to other genes as follow [24]:

gene _ score = abs(1 − 2 ∗ ϕ_(μ, σ)_ max(logFC)).

The edge weight between a source (S) and target (T) nodes was derived by the product of the S gene-score and T gene-score.

This weighted interaction network was used together with the seed gene list (list of differentially expressed genes) in PheNetic [23, 24]. PheNetic aims at connecting as many genes as possible from the seed list on the interaction network in the most parsimonious way (using the least number of edges). By enforcing such a parsimonious solution, PheNetic detects subnetworks in which genes from the seed list are closely connected. Such connected components can be viewed as proxies of pathways. The PheNetic *expression* subcommand was run in the downstream mode with the following parameters: min cost: 0.1; max cost: 5; step size: log scale between max and min cost with 28 steps; path-length = 4; k-best paths: 50; for all other parameters the default values were used. For each edge cost, the highest-scoring subnetwork was selected. Furthermore, the Jaccard index of all the subnetworks with the same edge cost was computed. For each cost, the subnetwork is rejected if it has a low stability score (i.e. Jaccard index smaller than 0.5) or if it is too large (more than 500 interactions). The final subnetworks are then the union of all the “best subnetworks” for each edge penalty that passed the stability and size requirements.

### Gene groups used for network analysis

The gene groups used for network analysis were derived from the Venn diagram displayed in Fig. 3. To perform network analysis, we defined respectively restoring genes (group A, genes with altered expression in the *bri1–5* mutant, but restored to WS2 level in at least 2 suppressors), compensatory genes (group B, (genes not differentially expressed in the *bri1–5* mutant, but differentially expressed in at least two suppressor strains), non-restored genes (group C, genes that differentially expressed in the *bri1–5* mutant and at least two of the suppressor strains) and genes that are uniquely affected in respectively the *bri1–5/brs1–1D* (group D), the *bri1–5/bak1–1D* (group F), and the *bri1–5/bri-1D* (group E) (Additional file 1).

To study the interaction between restored, compensatory, and non-restored genes, we combined the genes of groups A, B, and C (789 genes) to perform network analysis. Each group consists of its core genes. The core of group A (restored genes) consists of 118 genes that are restored to the wild-type state by all suppressors. The core of group B (compensatory genes) consists of 23 genes, i.e. genes that were significantly differentially expressed in all three *bri1–5* suppressors, but not in *bri1–5* mutant. The core of group C (non-restored genes) consists of 149 genes that are affected in *bri1–5* mutant but not restored by any suppressors. However, as the size of the gene sets is dependent on the choice of an arbitrary threshold, we assumed that some of the genes belonging to the processes represented by these core gene sets were found to be significantly differentially expressed in two of the three lines only (and slightly below the threshold in the third). That is why we extended the core gene sets with the genes that were differentially expressed in at least two lines, rather than in all three of them. Extended gene sets A, B and C were subsequently combined to study the interaction between the restored, compensatory, and non-restored genes.

## Supplementary Information

**Additional file 1.** List of differentially expressed genes as compared to WS2 in each line and group membership. (XLSM 104 kb)

**Additional file 2.** List of marker genes being differentially expressed upon addition of external BRs or in line with a gain of mutation in BR signaling genes at least in 5 studies.

**Additional file 3.** GO enrichment for genes exclusively differentially expressed in each suppressor.

**Additional file 4: Fig. S1.** The T-DNA insertion site for *bri1–1D* (A), and microscopic images of 7-day old hypocotyl cells for WS2 (B), *bri1–5* (C)*, bri1–5/bak1–1D (*D*), bri1–5/bri1–1D* (E)*, bri1–5/brs1–1D* (F). **Fig. S2.** PCA plot for assessing the reproducibility of the gene expression dataset. Samples taken from the same genotype are represented in the same color. The plot indicates high consistency between replicate samples as they are located close to each other when plotted on the first and second principal components. **Fig. S3.** RT-qPCR results for relative expression of selected genes and their corresponding values from microarray analysis. The values represent the log2 of relative expression (sample1/sample2). Rows indicate gene names and columns show the comparison between the indicated lines. Columns with pink header represent the RT-qPCR values, and columns with yellow header are microarray measurements. The red color on the heatmap indicates that the gene has been up-regulated in sample1 as compared to sample2, while blue indicates down-regulation. **Fig. S4.** Comparing genome-wide expression impact between *bri1–5* suppressor lines. **Fig. S5.** Heatmap of expression of the marker genes that up/down regulation of their expression was confirmed by at least 5 independent references and also affected in the *bri1–5* line of our study. For each line, the row-scaled normalized expression data of the 3 biological replicates are shown as adjacent columns. In each row the gradient red color indicates the higher expression for the gene compared to other samples while blue indicates the lower expression. **Fig. S6.** Pathway analysis (MapMan metabolism) showing for each mutant line the expression changes compared to WS2. Panel A: *bri1–5,* Panel B*: bri1–5/bri1–1D*, Panel C: *bri1–5/brs1–1D*, Panel D: *bri1–5/bak1–1D*. **Fig. S7.** Pathway analysis (MapMan: large enzyme families) showing for each mutant line the expression changes compared to WS2. Panel A: *bri1–5,* Panel B*: bri1–5/bri1–1D*, Panel C: *bri1–5/brs1–1D*, Panel D: *bri1–5/bak1–1D*. **Fig. S8.** Pathway analysis (MapMan: gene regulation) showing for each mutant line the expression changes compared to WS2. Panel A: *bri1–5,* Panel B*: bri1–5/bri1–1D*, Panel C: *bri1–5/brs1–1D*, Panel D: *bri1–5/bak1–1D*. **Fig. S9.** Expression pattern in each mutant line of genes related to ABA signaling, Glutathione metabolism, and ion related hemostasis as discussed in the main text. Mutant lines are represented in the x-axis. The y-axis indicates the log2 normalized expression value of the gene. **Table S1.** RT-qPCR test of log-fold change (log-FC) of the genes that are overexpressed by activation-tagging in the suppressors at the 7 days seedling stage. **Table S2.** Summary of the most significant results obtained by MapMan pathway analysis (metabolism, regulation and, large-enzyme families overview). Left column: enriched pathways; entries provide for each line the degree to which the pathway is enriched. *P*-values are FDR corrected using Benjamini-Hochberg). **Table S3.** Designed primers for RT-qPCR.

## Data Availability

The microarray datasets have been deposited in GEO under accession number GSE70843. Requests for resources, codes, and material should be directed to and will be fulfilled by the Lead Contact, Jia Li (lijia@lzu.edu.cn).

## Abbreviations

BR: Brassinosteroid
BRI: BR insensitive
BRL1: BRI1-like homologs
BAK1: BRI1-Associated receptor kinase
ABA: Abscisic acid
BIN: Brassinosteroid insensitive
BRZ: Brassinazole-Resistant
BES: Br-Insensitive-Ems-Suppressor
PP: Phosphatase
BRS: BRI suppressor
SCP: Serine carboxypeptidase
ECS: Extra Carpels and seeds
FLS: Flagellin-Sensitive
RT-qPCR: Real time quantitative polymerase chain reaction
PCA: Principal component analysis
ROS: Reactive oxygen species
FDR: False discovery rate
TF: Transcription Factor
FunTFBS: Functional transcription binding site
AtPIN: *Arabidopsis thaliana* protein interaction network
