# Supplementary material for: Genome-wide expression and network analyses of mutants in key brassinosteroid signaling genes
Source: BMC Genomics. 2021 Jun 22;22:465. doi: 10.1186/s12864-021-07778-w (PMC8220701; doi:10.1186/s12864-021-07778-w)
Supplement: Supplementary file 4 — Additional file 4: Fig. S1. The T-DNA insertion site for bri1–1D (A), and microscopic images of 7-day old hypocotyl cells for WS2 (B), bri1–5 (C), bri1–5/bak1–1D (D), bri1–5/bri1–1D (E), bri1–5/brs1–1D (F). Fig. S2. PCA plot for assessing the reproducibility of the gene expression dataset. Samples taken from the same genotype are represented in the same color. The plot indicates high consistency between replicate samples as they are located close to each other when plotted on the first and second principal components. Fig. S3. RT-qPCR results for relative expression of selected genes and their corresponding values from microarray analysis. The values represent the log2 of relative expression (sample1/sample2). Rows indicate gene names and columns show the comparison between the indicated lines. Columns with pink header represent the RT-qPCR values, and columns with yellow header are microarray measurements. The red color on the heatmap indicates that the gene has been up-regulated in sample1 as compared to sample2, while blue indicates down-regulation. Fig. S4. Comparing genome-wide expression impact between bri1–5 suppressor lines. Fig. S5. Heatmap of expression of the marker genes that up/down regulation of their expression was confirmed by at least 5 independent references and also affected in the bri1–5 line of our study. For each line, the row-scaled normalized expression data of the 3 biological replicates are shown as adjacent columns. In each row the gradient red color indicates the higher expression for the gene compared to other samples while blue indicates the lower expression. Fig. S6. Pathway analysis (MapMan metabolism) showing for each mutant line the expression changes compared to WS2. Panel A: bri1–5, Panel B: bri1–5/bri1–1D, Panel C: bri1–5/brs1–1D, Panel D: bri1–5/bak1–1D. Fig. S7. Pathway analysis (MapMan: large enzyme families) showing for each mutant line the expression changes compared to WS2. Panel A: bri1–5, Panel B: bri1–5/bri1–1D, Panel C: [file 12864_2021_7778_MOESM4_ESM.docx]

**Supplementary figures and tables**

**
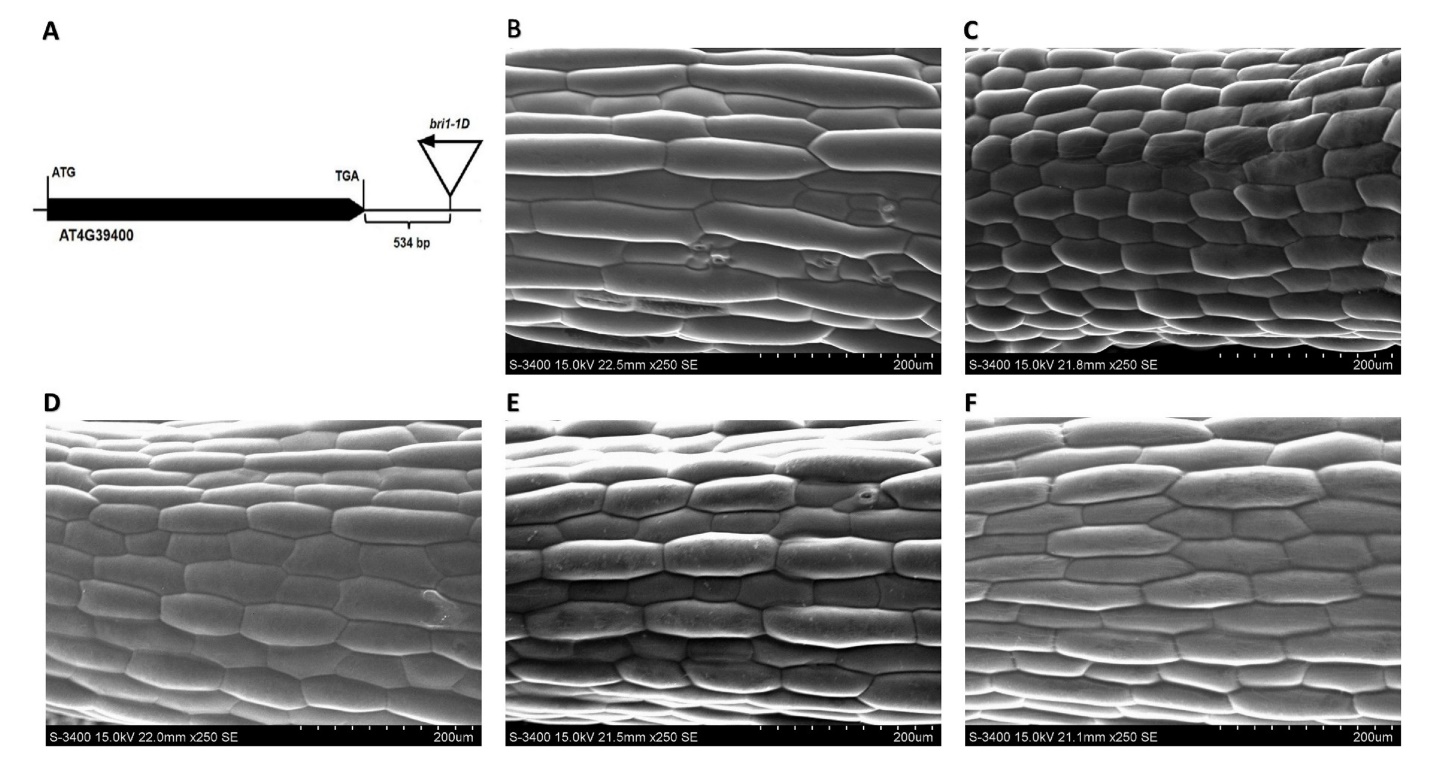
**

**Figure S1**: The T-DNA insertion site for *bri1-1D* (A), and microscopic images of 7-day old hypocotyl cells for WS2 (B), *bri1-5* (C)*,* *bri1-5/bak1-1D (*D*), bri1-5/bri1-1D* (E)*, bri1-5/brs1-1D* (F).


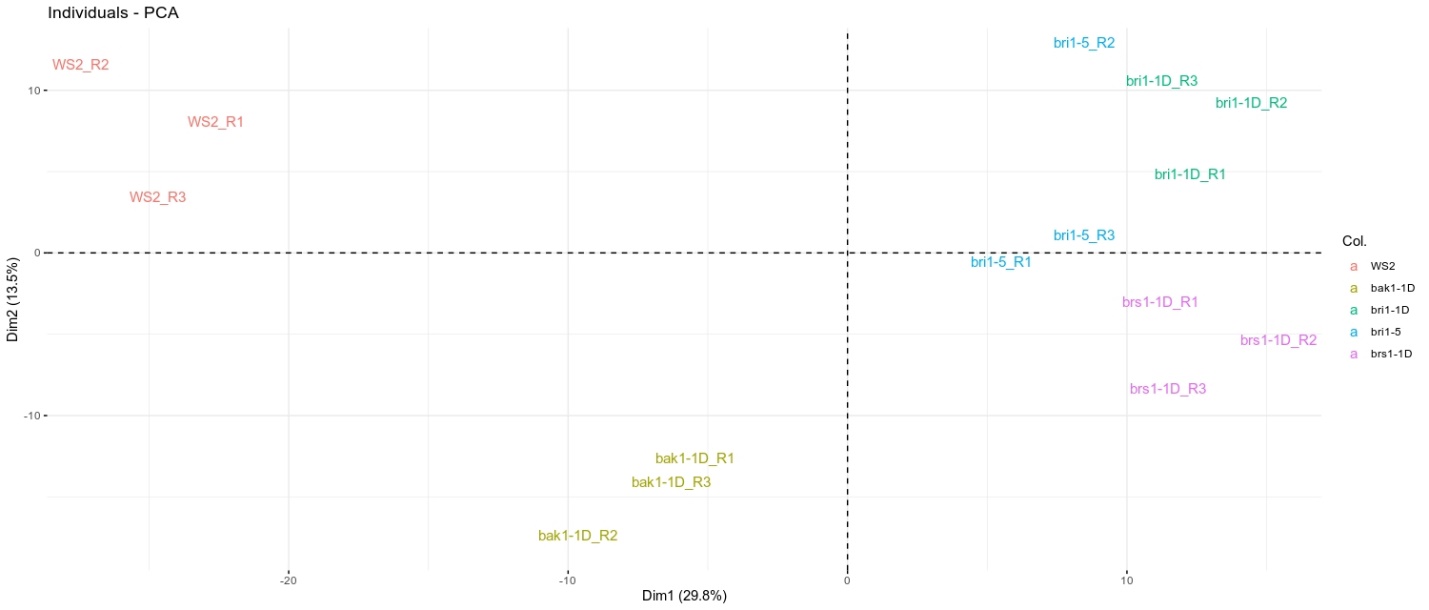


**Figure S2**: PCA plot for assessing the reproducibility of the gene expression dataset. Samples taken from the same genotype are represented in the same color. The plot indicates high consistency between replicate samples as they are located close to each other when plotted on the first and second principal components.


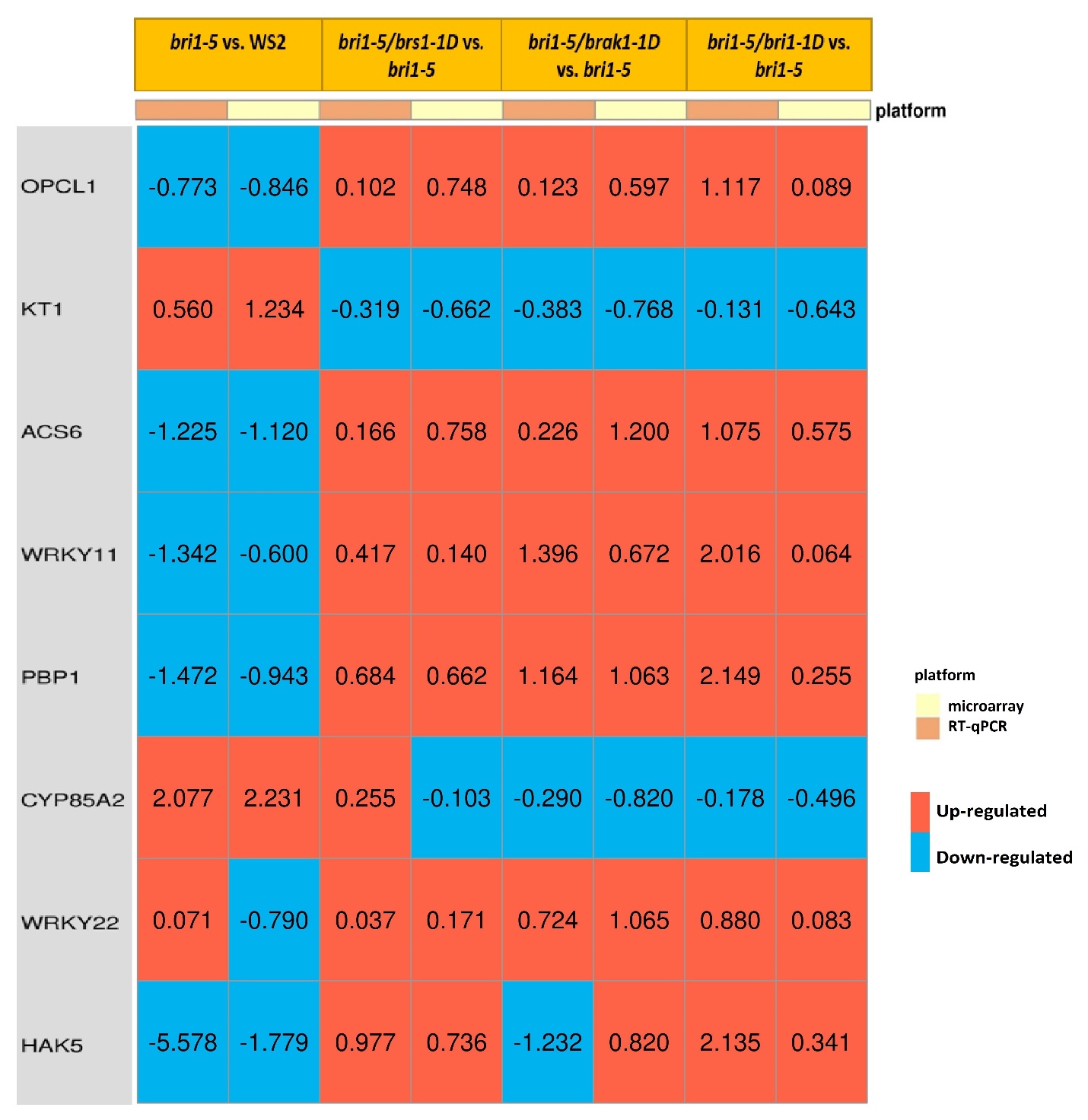


**Figure S3**: RT-qPCR results for relative expression of selected genes and their corresponding values from microarray analysis. The values represent the log2 of relative expression (sample1/sample2). Rows indicate gene names and columns show the comparison between the indicated lines. Columns with pink header represent the RT-qPCR values, and columns with yellow header are microarray measurements. The red color on the heatmap indicates that the gene has been up-regulated in sample1 as compared to sample2, while blue indicates down-regulation.


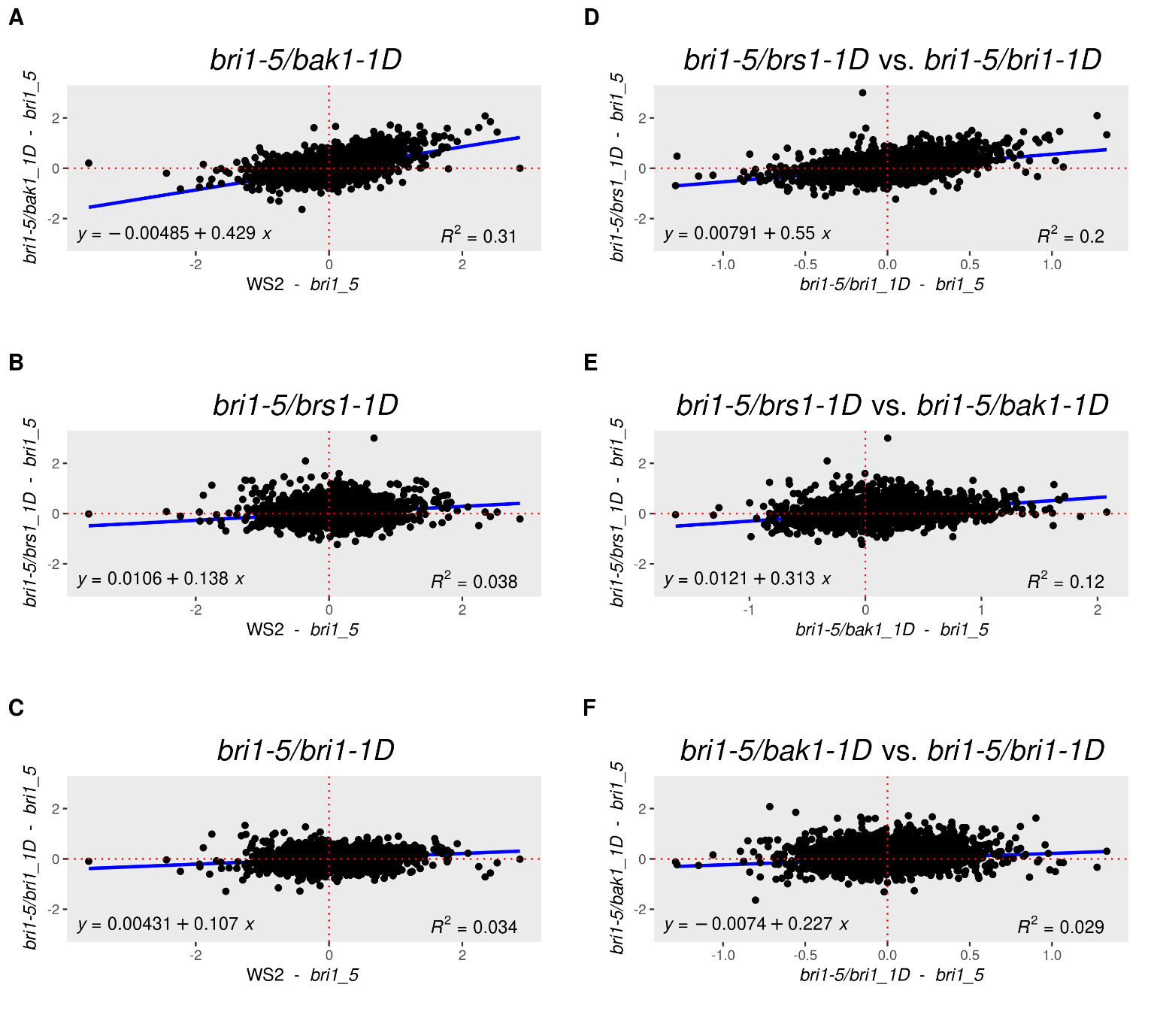


**Figure S4.** Comparing genome-wide expression impact between *bri1-5* suppressor lines. Panel A-C: Scatter plots display the log2 mean expression difference between each suppressor and *bri1-5* (Y-axis) versus the log2 mean expression difference of WS2 and *bri1-5* (X-axis). A value around zero on the Y-axis and a non-zero value on the X-axis means that a gene that was affected in the *bri1-5* mutant is not restored by the suppressor line displayed in the subplot title. A positive regression slope indicates that genes that are affected in the *bri1-5* versus WS2 have been restored to WS2 level by the suppressor line displayed on the subplot title. So it indicates that the aberrant expression observed in the *bri1-5* with the WS2 as a reference is restored for in the suppressor mutant. Hence, a stronger positive regression indicates that the suppressor mutant better approximates the WS2 and hence better compensates for the *bri1-5* phenotype. Panels D-F indicate to what extent the same genes were affected in the *bri1-5* suppressors lines (*bri1-5*/*bri1-1D*, *bri1-5*/*brs1-1D,* and *bri1-5*/*bak1-1D*) using the *bri1-5* as a common reference. A higher positive correlation (or regression slope) indicates that similar genes are affected by each pair of compared mutant lines.

**
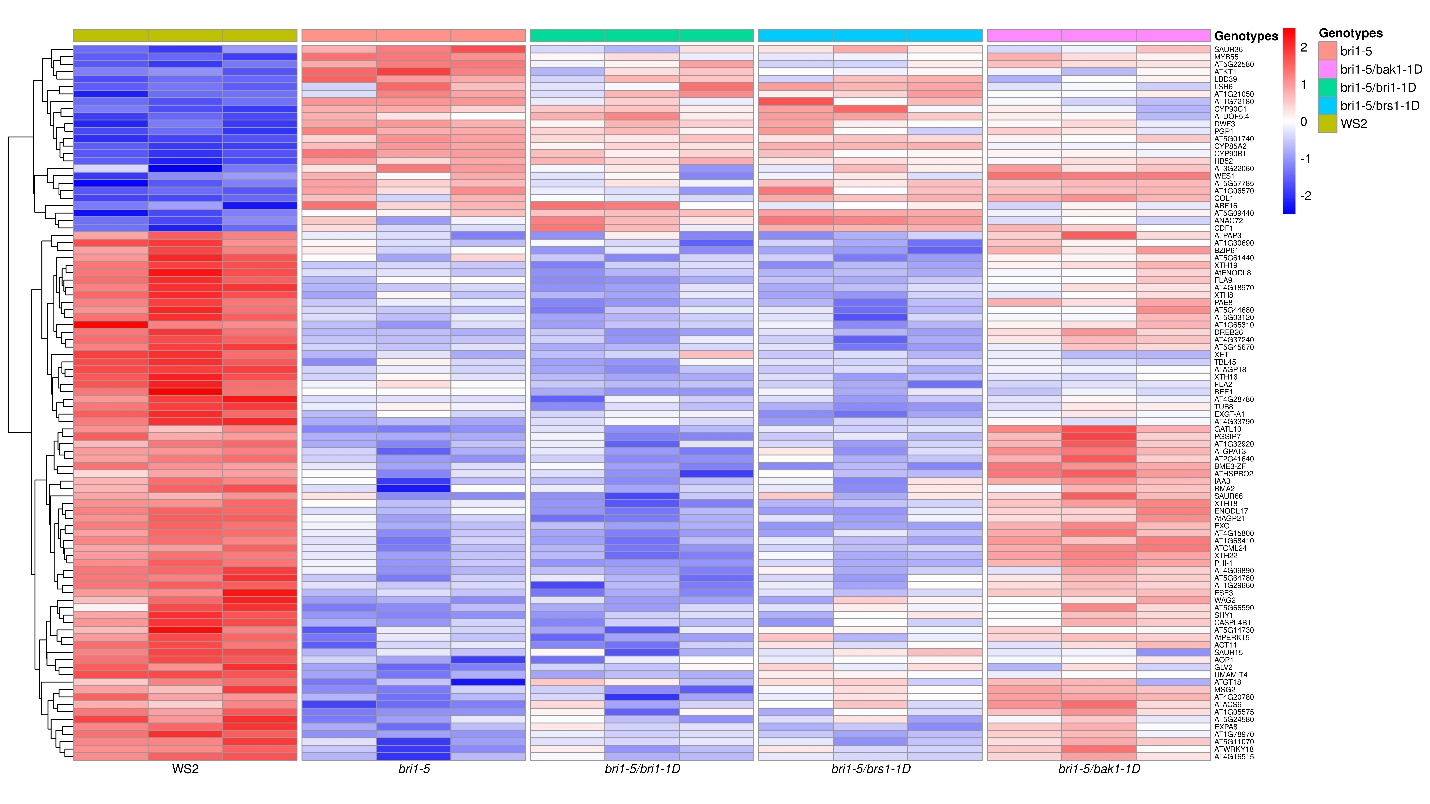
Figure S5:** Heatmap of expression of the marker genes that up/down regulation of their expression was confirmed by at least 5 independent references and also affected in the *bri1-5* line of our study. For each line, the row-scaled normalized expression data of the 3 biological replicates are shown as adjacent columns. In each row the gradient red color indicates the higher expression for the gene compared to other samples while blue indicates the lower expression.

**
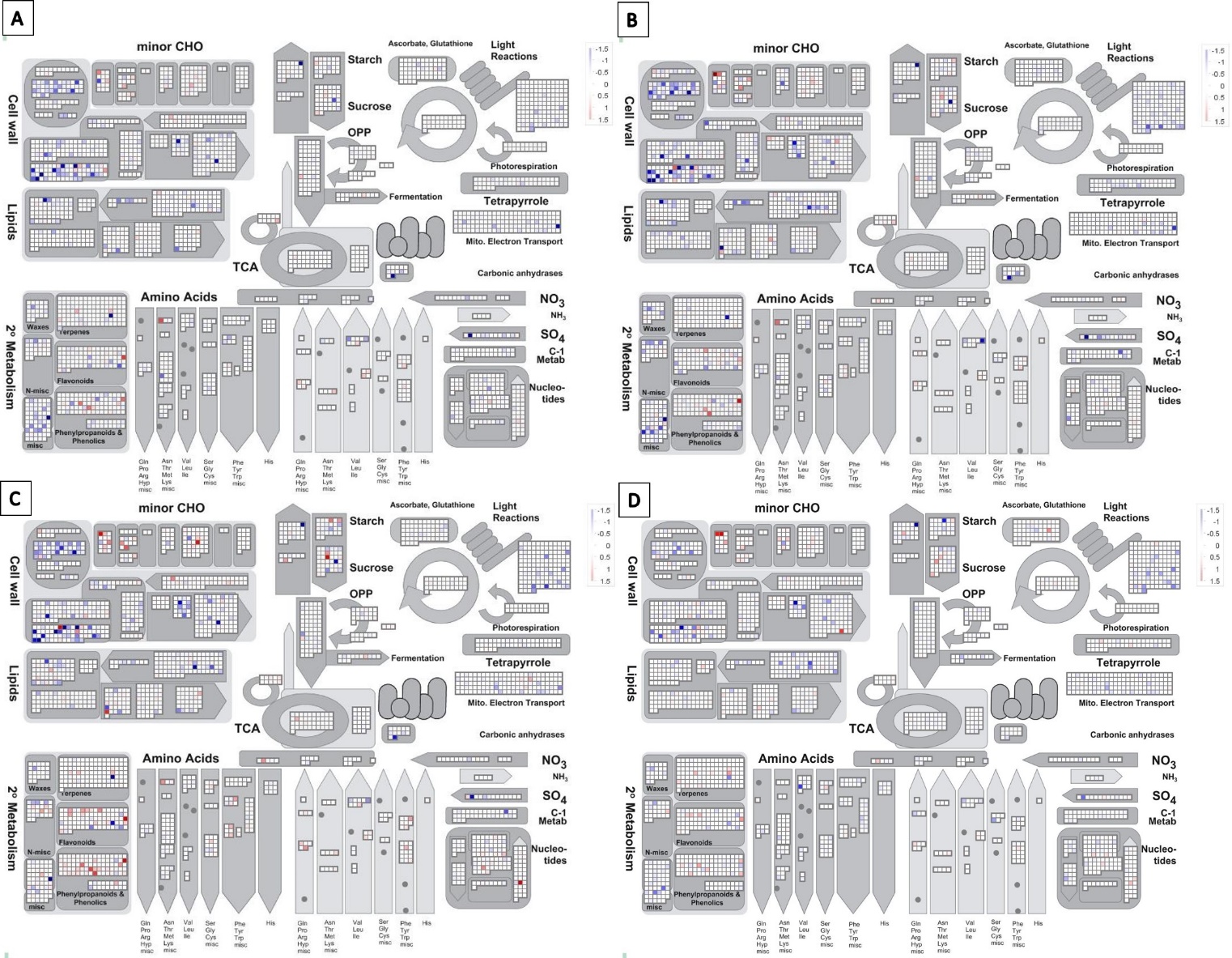
**

**Figure S6**: Pathway analysis (MapMan metabolism) showing for each mutant line the expression changes compared to WS2. Panel A: *bri1-5,* Panel B*: bri1-5/bri1-1D*, Panel C: *bri1-5/brs1-1D*, Panel D: *bri1-5/bak1-1D*.

**
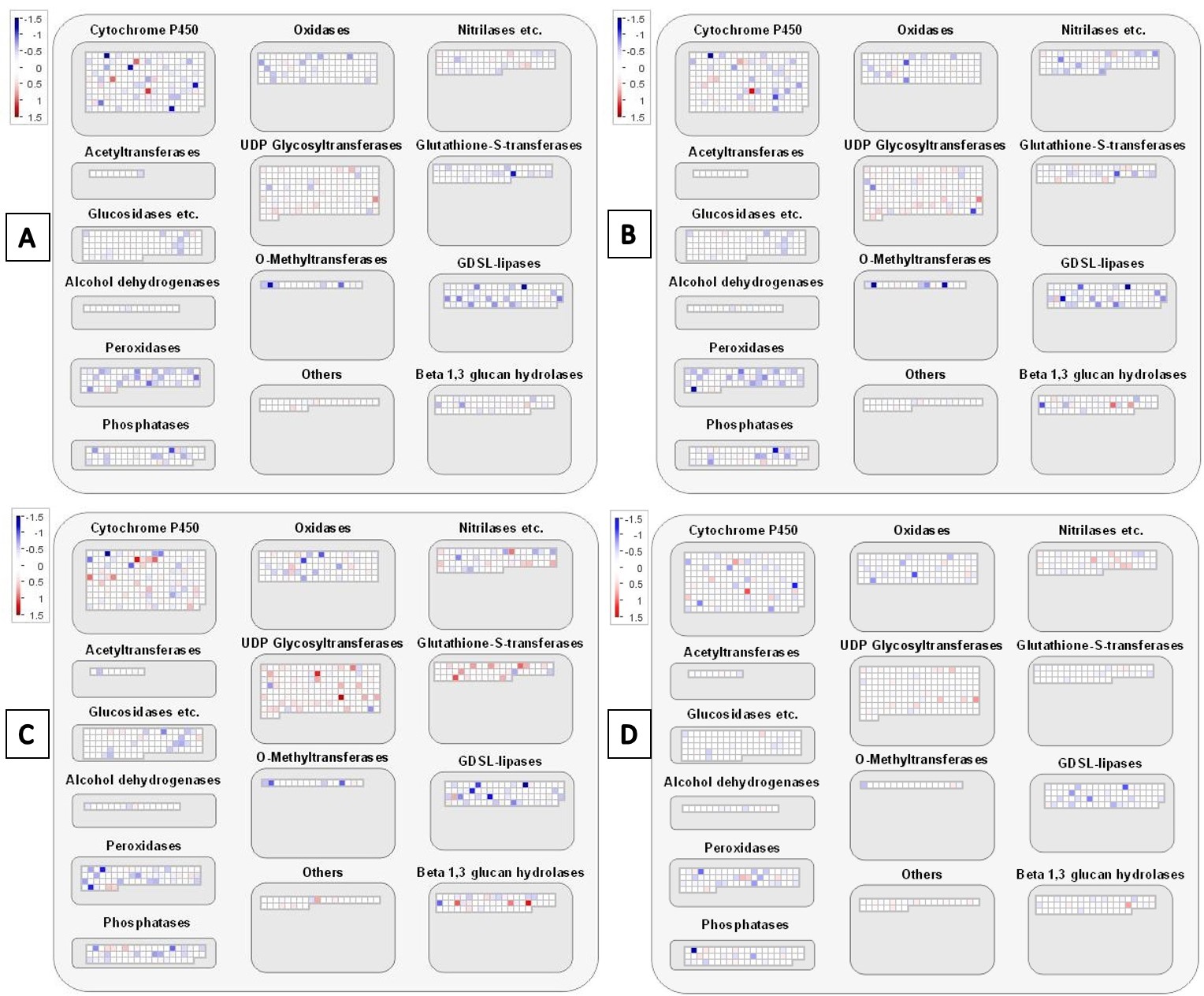
**

**Figure S7**: Pathway analysis (MapMan: large enzyme families) showing for each mutant line the expression changes compared to WS2. Panel A: *bri1-5,* Panel B*: bri1-5/bri1-1D*, Panel C: *bri1-5/brs1-1D*, Panel D: *bri1-5/bak1-1D*.

**
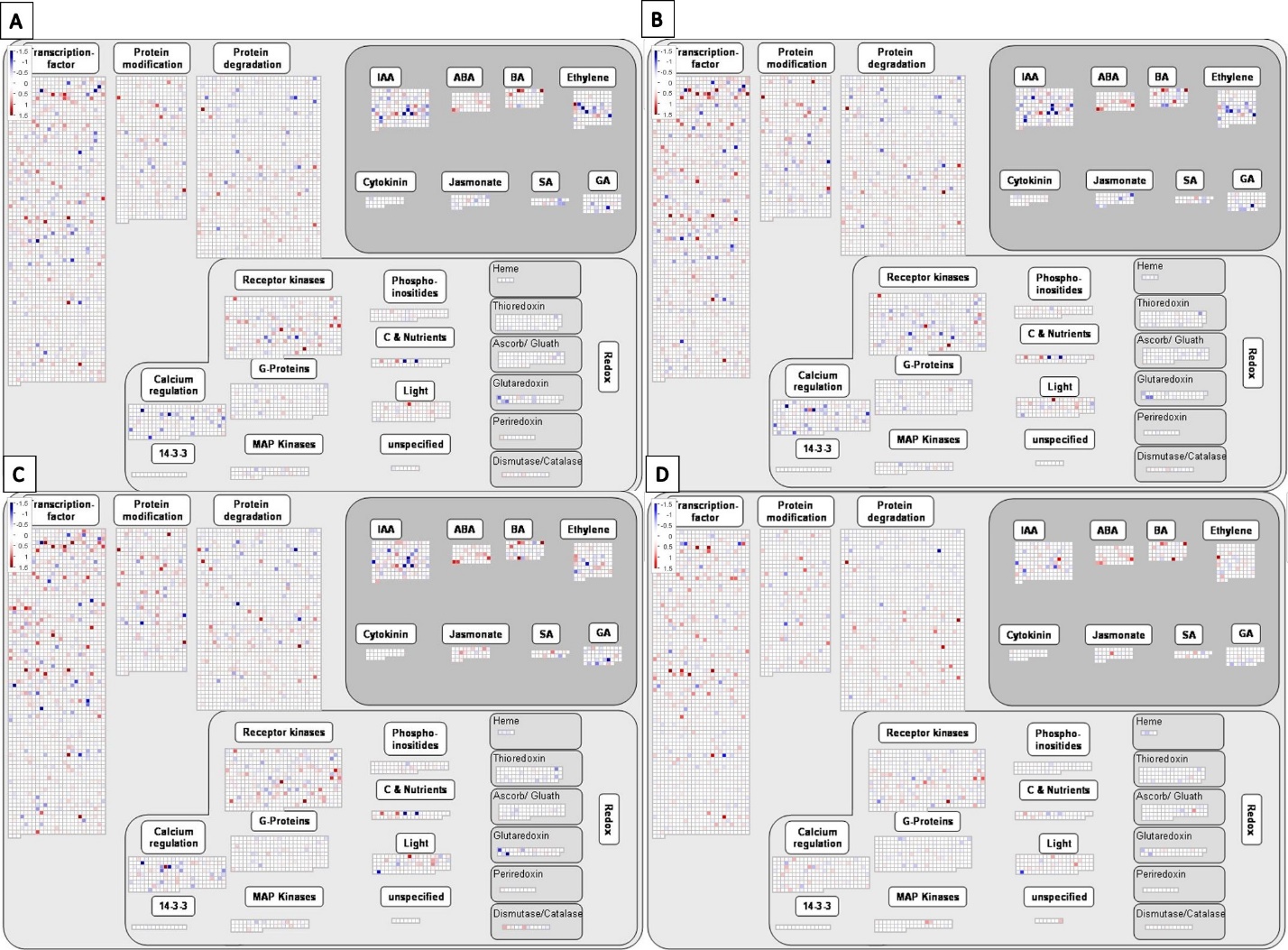
**

**Figure S8**: Pathway analysis (MapMan: gene regulation) showing for each mutant line the expression changes compared to WS2. Panel A: *bri1-5,* Panel B*: bri1-5/bri1-1D*, Panel C: *bri1-5/brs1-1D*, Panel D: *bri1-5/bak1-1D*.


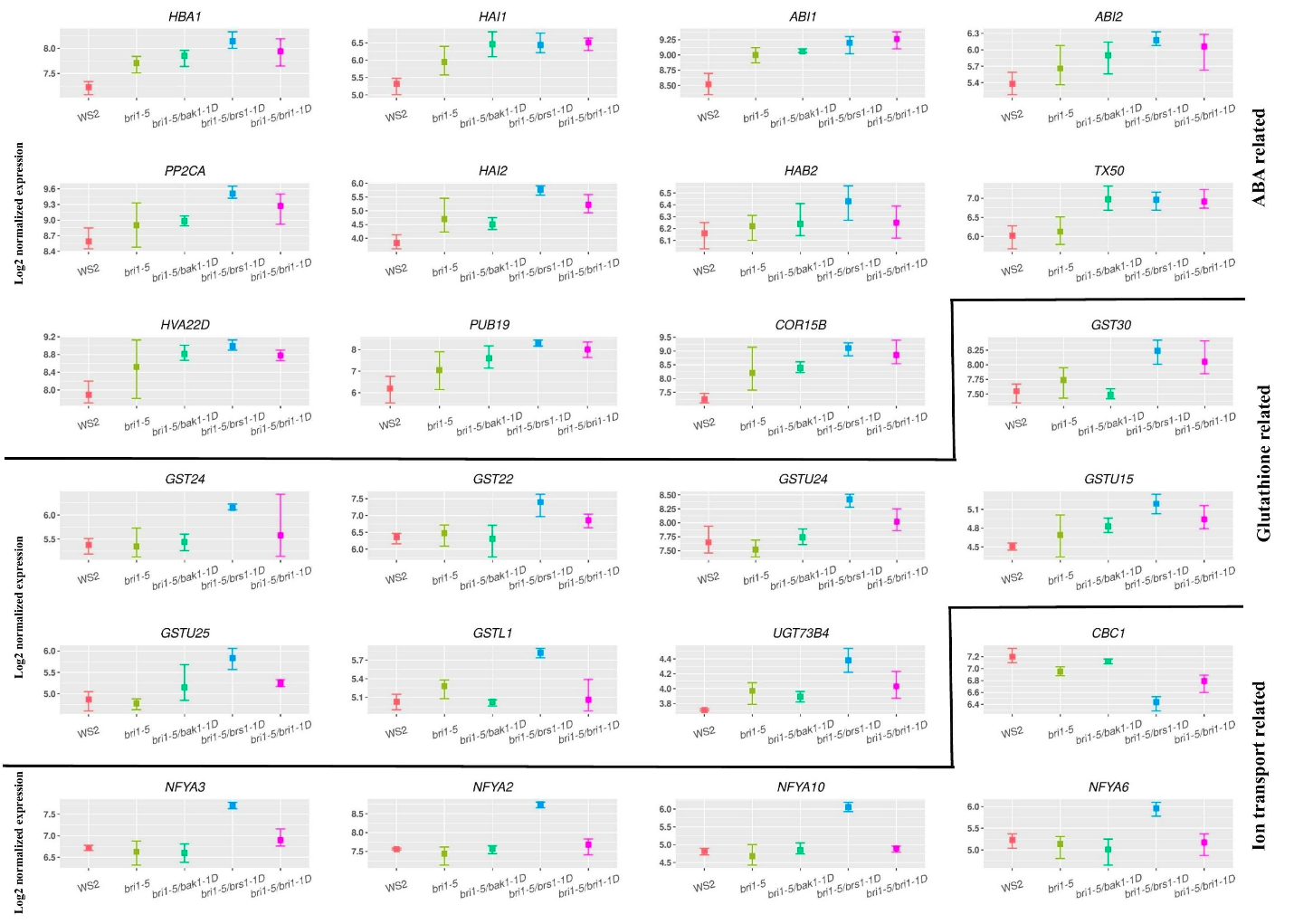


**Figure S9**: Expression pattern in each mutant line of genes related to ABA signaling, Glutathione metabolism, and ion related hemostasis as discussed in the main text. Mutant lines are represented in the x-axis. The y-axis indicates the log2 normalized expression value of the gene.

**Table S1**. RT-qPCR test of log-fold change (log-FC) of the genes that are overexpressed by activation-tagging in the suppressors at the 7 days seedling stage.

|  | Log-FC (RT-qPCR) | Log-FC (microarray) |
| --- | --- | --- |
| BAK1 (*bri1-5/bak1-1D* vs. *bri1-5*) | 3.576 | 1.611 |
| BRI1 (*bri1-5/bri1-1D* vs. *bri1-5*) | 1.847 | 0.911 |
| BRS1 (*bri1-5/brs1-1D* vs. *bri1-5*) | 2.388 | 5.386 |

**Table S2:** Summary of the most significant results obtained by MapMan pathway analysis (metabolism, regulation and, large-enzyme families overview). Left column: enriched pathways; entries provide for each line the degree to which the pathway is enriched. P-values are FDR corrected using Benjamini-Hochberg).

|  | *bri1-5* | *bri1-5*/*bri1-1D* | *bri1-5*/*brs1-1D* | *bri1-5*/*brak1-D* |
| --- | --- | --- | --- | --- |
| RNA regulation of transcription | 1e-20 | 1e-20 | 9.10e-14 | 1e-20 |
| Protein degradation | 6.77e-7 | 1.45e-9 | 7.46-3 | 6.17e-10 |
| Protein post-ranslational modification | 1.02e-4 | 5.46e-3 | 7.06e-2 | 3.13e-3 |
| Amino acid metabolism | 2.13e-2 | 2.71e-3 | 1.34e-3 | 1.39-5 |
| Cell wall | 2.01e-12 | 1.25e-11 | 5.97e-7 | 2.25e-2 |
| Mitochondrial electron transport- / ATP synthesis | 1.48e-8 | 3.63e-6 | 1.14e-5 | 2.69e-8 |
| Lipid metabolism- FA synthesis and FA elongation | 1.423-3 | 3.69e-3 | 2.04e-3 | 7.79e-7 |
| Secondary metabolism- sulfur-containing | 4.30e-3 | 1.17e-5 | 7.17e-2 | 2.63e-5 |
| Cell wall- cell wall proteins (AGPs) | 7.26e-9 | 7.16e-9 | 8.64e-9 | 9.83e-4 |
| PS- light reaction | 1.07e-8 | 1e-20 | 1.36e-11 | 1.56e-2 |
| Cell wall- cellulose synthesis | 9.30e-2 | 1.67e-3 | 4.63e-4 | 9.01e-2 |
| Abscisic acid (ABA) metabolism | 0.28 | 3.25e-2 | 7.11e-2 | 4.53e-5 |
| Cytochrome P450 | 3.35e-4 | 0.87 | 0.72 | 0.34 |
| Glutathione S transferases | 8.87e-4 (down-regulated genes) | 0.12 | 2.48e-6 (up-regulated genes) | 0.41 |

**Table S3.** Designed primers for RT-qPCR.

| Gene name | Gene symbol | Forward primer | Reverse primer |
| --- | --- | --- | --- |
| Actin | ACT | TCAGATGCCCAGAAGTCTTGTTCC | CCGTACAGATCCTTCCTGATATCC |
| AT1G20510 | OPCL1 | TCAAATCCAGTCCTCGCCTT | TCGTTCCTCGTCCATAAGCA |
| AT1G80840 | WRKY40 | AGGGATCAAATCAGCCCTCC | GAGCTACTCTCCGACACTCC |
| AT2G30070 | KT1 | TGGAACCCGCGTATAGTCTC | ACACCTCCAAGTGAAACCCA |
| AT3G30180 | BR6OX2 | GAAAGGACTTGTTGCCGGTT | CAACGAGCCTCTCATTAGCC |
| AT3G49580 | LSU1 | TTAAGTTGTGGCAGCGAACG | AGAGCATGCGATCGTGATAGT |
| AT4G01250 | WRKY22 | TCTTTGTCGGATACGGTGGT | AGAGATCAAAACTCGCCGGA |
| AT4G11280 | ACS6 | TTTCGTCACAAACGCAGCAT | GGTTATCTCAGCGTGCCTTG |
| AT4G13420 | HAK5 | GACCGAGATTACGGCAGAGA | CGCAAGTGCTTTGTCTCCTT |
| AT4G31550 | WRKY11 | CCACCGTCTAGTGTAACACTCGAT | TGCAACGGAGCAGAAGCAAGGAA |
| AT5G54490 | PMP1 | TTGCAAAGGGTTCGAGCTTC | TATCGAACATCGTCGTCCGT |
| \|  \| [AT4G33430](https://www.arabidopsis.org/servlets/TairObject?id=127207&type=locus) \| \| --- \| --- \| | BAK1 | AGACTGGGTGAAAGGGTTGT | TAGCTGCTCCACTTCTTCGT |
| \|  \| [AT4G39400](https://www.arabidopsis.org/servlets/TairObject?id=26531&type=locus) \| \| --- \| --- \| | BRI1 | TTTGCACGACCCCAAGAAAG | TGTGGTGAAGGAAAGCAAGC |
| \|  \| [AT4G30610](https://www.arabidopsis.org/servlets/TairObject?id=127117&type=locus) \| \| --- \| --- \| | BRS1 | TCACGGTGGAGAGAGTTTCC | CGTTGGGGTGTAAATGCTGT |
